# Taste receptor downregulation in diet-induced obesity reveals circumvallate papillae-specific taste-inflammatory networks

**DOI:** 10.3389/fnut.2026.1857562

**Published:** 2026-06-24

**Authors:** Isabella Kimmeswenger, Andrej Feješ, Veronika Somoza, Katarína Šebeková, Barbara Lieder

**Affiliations:** 1 Faculty of Chemistry, Institute of Physiological Chemistry, University of Vienna, Vienna, Austria; 2 Vienna Doctoral School in Chemistry (DoSChem), University of Vienna, Vienna, Austria; 3 Faculty of Medicine, Institute of Molecular Biomedicine, Comenius University, Bratislava, Slovakia; 4 Christian Doppler Laboratory for Taste Research, Faculty of Chemistry, University of Vienna, Vienna, Austria; 5 Institute of Clinical Nutrition, University of Hohenheim, Stuttgart, Germany

**Keywords:** cafeteria diet, extra-oral taste receptors, fungiform papillae, inflammation, sweet taste receptor

## Abstract

**Introduction:**

Beyond their role in chemosensory perception, taste receptors function as metabolic regulators extra-orally. While decreased taste receptor expression in gustatory tissue is linked to obesity-associated inflammation, we tested the hypothesis that taste receptor-inflammation interactions extend to extra-oral contexts.

**Methods:**

Obesity-associated metabolic syndrome-like phenotype was induced in female C57BL/6 mice by administration of a cafeteria diet for 18 weeks. The controls consumed a standard diet. Gene-expression levels of *Tas1r2*, *Tas1r3*, *CD36*, *Ffar1*, *GPR84* and *Ffar4* were analyzed in circumvallate papillae, lower jejunum, stomach and spleen using RT-qPCR. The number and area of fungiform papillae were assessed on the anterior tongue. Correlation network connectivity for taste receptor expression and fungiform papillae markers with inflammatory and cardiometabolic risk markers was calculated tissue-specifically.

**Results and discussion:**

The cafeteria diet induced obesity, metabolic dysfunction, and systemic inflammation. Additionally, number and area of fungiform papillae were reduced, and *Tas1r2, CD36, Ffar1* and *GPR84* were downregulated in circumvallate papillae, whereas in jejunum only *CD36*, *Ffar1,* and *GPR84* were downregulated following cafeteria diet. Gustatory sweet and fatty acid receptor expression correlated with dietary intake patterns. Exploratory network analysis indicated tissue-specific correlation patterns, with taste receptor-inflammation crosstalk predominantly appearing orally. In contrast, extra-oral sweet and fatty acid receptor expression showed weaker or absent correlations with systemic inflammatory markers under the conditions tested.

## 1 Introduction

Taste receptors, primarily studied for their role in gustatory perception, have gained increasing recognition for their importance as nutrient sensors and metabolic regulators beyond the oral cavity (1). Among these, the sweet taste receptor, consisting of the two subunits taste 1 receptor member 2/3 (TAS1R2/TAS1R3), and fatty acid receptors such as fatty acid translocase (CD36), free fatty acid receptor 1 (Ffar1), G-protein-coupled receptor 84 (GPR84), and free fatty acid receptor 4 (Ffar4) are of particular relevance regarding energy homeostasis, as they are involved in glucose homeostasis, insulin signaling, and lipid metabolism (2–6). The sweet taste receptor subunits can be found in the small intestine, where they have been implicated in glucose metabolism (7–9). *In vitro* studies using enteroendocrine cell models demonstrated that activation of TAS1R3 leads to glucagon-like peptide 1 (GLP-1) release (7, 8) and activation of TAS1R2 and TAS1R3 to glucose-dependent insulinotropic polypeptide (GIP) secretion (8). The incretin hormones GLP-1 and GIP not only promote insulin secretion (10), but also enhance glucose uptake efficiency via an upregulation of SGLT-1 (7, 11). An upregulation of SGLT-1 is also mediated by TAS1R3 activation directly (8). Moreover, intestinal *TAS1R2* expression increases in response to luminal glucose levels under euglycemic conditions but decreases during hyperglycemia in healthy individuals. This mechanism appears to be impaired under diabetic conditions, as *TAS1R2* expression was shown to be increased during hyperglycemia (9), raising questions about the role of the intestinal sweet taste receptor in metabolic disease.

Whereas the sweet taste receptor subunits TAS1R2 and TAS1R3 are involved in glucose metabolism, fatty acid receptors play a role in the regulation of lipid metabolism (12, 13). While in taste cells, these receptors mediate sensory transduction upon fatty acid binding (14), they also regulate lipid uptake of enterocytes, with *CD36*, *Ffar1*, and *Ffar4* mRNA expression correlating positively with dietary fat content to facilitate absorption. Receptor activation simultaneously triggers cholecystokinin (CCK) and peptide YY (PYY) secretion, coordinating fat digestion and transmitting satiety signals to hypothalamic centers, regulating energy homeostasis (12, 15, 16). Consequently, diminished fatty acid receptor expressions in gastrointestinal tissues could impair satiety signaling and promote excessive caloric intake.

Dietary interventions have been shown to modulate taste receptor gene expression in gustatory tissue. Caloric restriction increased gene expression of taste-related genes, including *Tas1r2* and *Tas1r3* in circumvallate papillae (CV) (17), while consumption of a Western diet with a sugar-sweetened beverage led to upregulation of bitter taste receptors and altered expression of markers for sweet-sensing receptors in CV (18). Diet-induced obesity further affected taste receptor gene expression, with high-fat diet (HFD) feeding resulting in reduced gustatory *Tas1r3* expression (19). Previously, obesity-associated chronic inflammation has been directly linked to structural changes in taste tissue, as Kaufman et al. demonstrated that chronic low-grade inflammation brought on by obesity reduced taste bud numbers in the gustatory tissue of mice by disrupting the balance between taste cell renewal and cell death (20). These morphological findings are supported by molecular evidence from Archer et al., who used RNA sequencing of human fungiform papillae (FP) to show that inflammation- and immune-response genes are upregulated in obese individuals, while taste-related genes are consistently downregulated (21). However, decreased taste receptor expression in obesity is not limited to gustatory tissue. Herrera et al. reported decreased sweet and bitter receptor gene expression in the duodenum of rats with HFD-induced obesity (22), suggesting that extra-oral taste receptors are also affected. However, whether extra-oral taste receptors show similar associations to inflammatory markers as in gustatory tissue remains unclear. Given that extra-oral taste receptors are involved in various aspects of energy metabolism, investigating their regulatory patterns is highly relevant. In the present study, we therefore examined correlation networks between sweet and fatty acid receptor expression and selected inflammatory and cardiometabolic markers across multiple tissues.

For this purpose, we induced obesity-related metabolic complications using a standardized protocol of cafeteria diet (CAF) as described by Lalanza and Snoeren (23). The cafeteria diet consists of multiple palatable food items with diverse tastes, textures, and aromas enabling voluntary self-selection, thus mimicking the human Western-style diet. Four menus are rotated every 2 days to maintain novelty and promote sustained hyperphagia (23). In preclinical studies, diet-induced obesity is most commonly modelled by feeding an HFD, which provides limited chemosensory stimulation compared with a varied food environment that characterizes human Western-style diet-associated overconsumption patterns (24). We here used the CAF mouse model to examine correlations between diet-induced inflammation markers and sweet and fatty acid receptor expression, as well as cardiometabolic and morphometric parameters in selected oral and extra-oral tissues. The chosen tissues included the circumvallate papillae (CV), representing primary taste signaling; lower jejunum and stomach involved in nutrient absorption and metabolic signaling; and spleen for systemic inflammatory responses (25, 26). We hypothesized here that obesity-associated inflammatory markers are associated with altered taste receptor gene expression independent of the analyzed tissue.

## 2 Materials and methods

### 2.1 Study design and experimental mouse model

All experimental procedures were approved by the Ethics Committee of the Institute of Pathophysiology (07/2023), Comenius University, Bratislava, State Veterinary and Food Administration of the Slovak Republic (4,503/2024-220), and conducted according to EU Directive 2010/63/EU and ARRIVE guidelines (27).

Six-month-old female C57BL/6 mice (*n* = 13) were obtained from The Jackson Laboratory (*Maine, USA*) and group-housed (2–3 per cage) in an open cage system (20 × 12 × 20 cm) under controlled conditions (temperature: 24 ± 2 °C, humidity: 55 ± 10%, 12-h light/dark cycle) with *ad libitum* access to a standard diet (*Ssniff V1534-000 R/M-H maintenance diet, Ssniff Spezialdiäten GmbH, Germany*) and water. After a 14-day-long quarantine, mice were divided into two groups of similar body weights (*p* = 0.363). The control group (CTRL; *n* = 7) continued on a standard diet, while the experimental group (*n* = 6) received a CAF diet. The sample size was calculated to detect a minimum 20% difference in body weight, with a significance level of *α* = 0.05 and statistical power (1 − *β*) = 0.80.

The CAF diet consisted of four rotating menus containing sweet, salty, and processed foods with varying textures (soft, crunchy, smooth, rigid, chewy) alongside standard chow, following established protocols (23) (Supplementary Table S1). Menus were changed every 2 days. The mice received the assigned diets for 18 weeks, corresponding to an intake from young adulthood to middle age in humans. Body weight was monitored weekly. To assess caloric intake, macronutrient consumption, and individual dietary preferences, mice were single-housed for 2 weeks prior to sacrifice. The first 6 days of individual housing served as a habituation period to ensure that body weight gain remained comparable to that observed under group-housed conditions, suggesting that single housing did not affect feeding behavior. Thereafter, measurements were conducted over 8 days to capture one complete cycle of all four menus.

### 2.2 Sacrifice and sampling

After overnight food deprivation, fasting blood glucose was measured from a tail snip using a glucometer (*Accu-Chek Performa, Roche Slovakia, Diabetes Care, Bratislava, Slovakia*). Thereafter, blood was collected under inhalation anesthesia (97% oxygen, 3% isoflurane) from the orbital plexus into EDTA and heparin tubes (*Microvette, Sarstedt, Nümbrecht, Germany*), and cervical dislocation was performed. Blood count was determined (*Abacus VET 5 Hemoanalyser, Diatron MI ZRT, Budapest, Hungary*), while remaining blood was centrifuged (1,600 × *g* at 4 °C for 10 min), plasma was aliquoted and stored at −20 °C for further analyses. Waist circumference and nose-to-anus length were measured, perigonadal and retroperitoneal fat masses were removed and weighed. Waist-length ratio, and relative abdominal fat mass were calculated. The stomach and lower jejunum (0.5 cm from the distal end of the jejunum) were rinsed with PBS. Tongues, jejunum, stomach and spleen were stored at −80 °C until processing.

### 2.3 Measurement of cardiometabolic and inflammatory parameters in plasma

Plasma triacylglycerols (TAG), total cholesterol and high-density lipoprotein cholesterol (HDL-C) were measured using a Biolis 24i Premium analyser (*Tokyo Boeki Machinery, Tokyo, Japan*). Non-HDL-C was calculated by subtracting HDL-C from total cholesterol. Fasting insulin concentration was determined by ELISA (*Mouse Insulin ELISA, Mercodia, Uppsala, Sweden*). The quantitative insulin sensitivity check index (QUICKI) was calculated as: 1/[log(fasting insulin, μIU/mL) + log(fasting glucose, mg/dL)] (28). Inflammatory cytokine concentrations (IL-1α, IL-1β, IL-6, IL-10, IL-17A, IL-23, IL-27, TNF-α, GM-CSF, MCP-1, IFN-γ, IFN-β) were analyzed using the LEGENDplex™ Mouse Inflammation Panel (13-plex) on a DxFlex cytometer (*Beckman, Indianapolis, Indiana, USA*) following manufacturer’s instructions.

### 2.4 Tissue preparation

Frozen tongues were cut in half, with the anterior part stored for FP analysis while the posterior part was incubated for 16 h in pre-cooled RNAlater-ICE (*Invitrogen, ThermoFisher Scientific, Vienna, Austria*) at −20 °C. The CVs were dissected, snap-frozen in liquid nitrogen, and homogenized in 1X DNA/RNA Protection Reagent (*New England Biolabs GmbH, Frankfurt am Main, Germany*) using a micro pestle. For the lower jejunum, stomach, and spleen, 10 mg of tissue were weighed and homogenized in 1X DNA/RNA Protection Reagent (*New England Biolabs GmbH, Frankfurt am Main, Germany*) using a micro pestle. Further lysis of all homogenates was ensured by passing through a syringe needle (20G × 1 1/2; 0.90 × 40 mm). Homogenates were centrifuged to remove debris, and the supernatant was stored at −80 °C until RNA isolation.

### 2.5 Gene expression analysis of taste and inflammatory markers

Gene-expression analysis was carried out as recently described in detail (17, 18). In brief, RNA extraction from tissues was performed using the Monarch Total RNA Miniprep Kit (*New England Biolabs GmbH, Frankfurt am Main, Germany*) according to the tissue sample protocol provided by the manufacturer. Homogenized tissues were incubated with Proteinase K, genomic DNA contamination was eliminated through DNase I treatment, and RNA purity was assessed photometrically by measuring absorbance ratios at 260/280 and 260/230 nm using a NanoQuant Plate of a Spark Tecan Plate Reader (*Tecan, Menningen, Switzerland*). Isolated RNA was converted to cDNA using the LunaScript RT Supermix Kit (*New England Biolabs GmbH, Frankfurt am Main, Germany*). Quantitative PCR reactions were performed in triplicate using Luna Universal qPCR Mastermix (*New England Biolabs GmbH, Frankfurt am Main, Germany*) on a Bioer’s LineGene 9660 Real-Time PCR Detection System (*Bioer Technology, Hangzhou, China*). LinReg PCR software (*Version 2021.2*) was used to calculate hypothetical mRNA starting concentrations (29). Expression levels were normalized to the geometric mean of three housekeeping genes (*Actb, Hprt1, 18s*). All primers were selected based on previous publications, synthesized by Sigma Aldrich Austria (*Merck Life Science, Vienna, Austria*), and validated through product sequencing (*Eurofins Genomics AT GmbH, Vienna, Austria*). Primer sequences, applied concentrations, and sources are listed in Supplementary Table S2.

### 2.6 Number and size of FP

FP number and size were assessed using a tongue observation method adapted from Mistretta et al. (30), with detailed procedures described previously (18). In brief, anterior tongue sections were stained using 0.5% (w/v) methylene blue solution, allowing visual differentiation of the lighter-stained FP from filiform papillae. Under a dissecting microscope (*Eschenbach Optik GmbH, Nürnberg, Germany*), FP anterior to the intermolar eminence were counted in duplicate by a single investigator. For size measurements, photographs of the anterior tongue were taken at 5× magnification using an Axio Lab A1 microscope (*Carl Zeiss, Jena, Germany*) equipped with an Axiocam ERc 5 s camera (*Carl Zeiss, Jena, Germany*). Mean papillae area was determined using Zen 2.3 Lite Software (*Carl Zeiss, Jena, Germany*) and ImageJ (*Version 1.8.0.322*).

### 2.7 Network analysis

For network analysis, all variables were standardized using a *z*-score transformation to enable comparison across different measurement scales. Pairwise correlations between all variables were calculated using Spearman’s rank correlation coefficient, accounting for the small sample size of *n* = 6–7 per group. To address multiple testing, *p*-values were corrected using the false discovery rate (FDR) method of Benjamini and Hochberg (31). Only correlations meeting both statistical significance (FDR-adjusted *p* < 0.05) and effect size criteria (│*r*│ > 0.5) were included in network construction. Cross-domain correlation networks were constructed to specifically examine relationships between taste-related variables (*Tas1r2*, *Tas1r3*, *CD36*, *Ffar1*, *GPR84*, *Ffar4*, FP number and area) and all other measured parameters (inflammatory receptors, cytokines, morphometric and cardiometabolic markers). This approach allowed the analysis of biologically relevant taste-metabolism crosstalk while excluding within-category correlations (e.g., cytokine–cytokine correlations). Node centrality measures were calculated to identify hub variables with the highest number of significant connections. All network analyses were performed separately for each tissue (CV, lower jejunum, stomach, and spleen) to assess tissue-specific patterns of taste-inflammation relationships. Network density was assessed against chance expectation using permutation testing (1,000 permutations with independent variable shuffling). A supplementary edge table reporting all cross-domain pairs with *r*, raw and FDR-adjusted *p*-values, and pairwise sample sizes is provided in Supplementary Tables S3–S6. Network analysis was conducted using RStudio (*Version 2025.05.1*) with the packages *igraph*, *ggraph*, and *tidygraph*.

### 2.8 Statistical analysis

**Figure 1:**
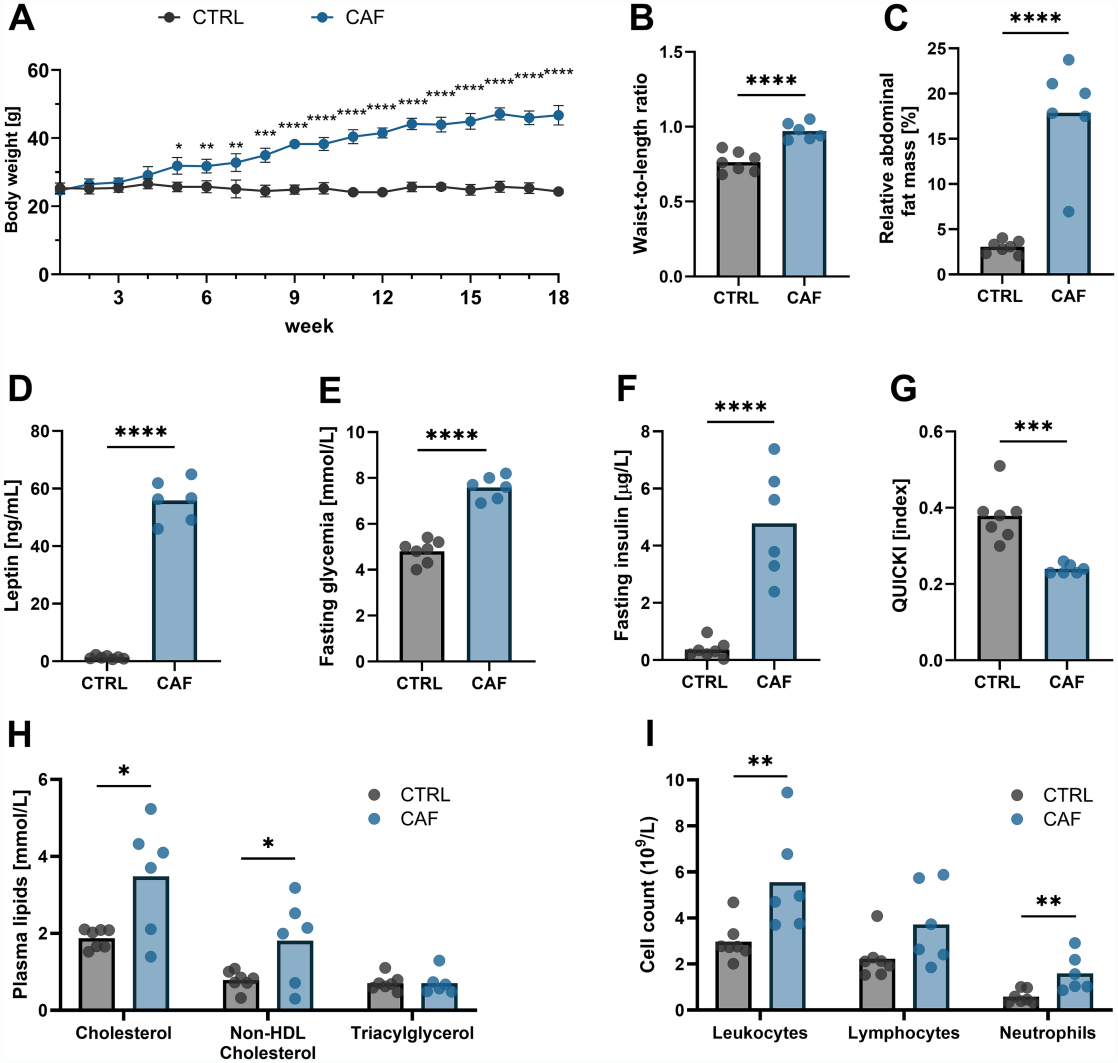
Morphometric and cardiometabolic markers. **(A)** Body weight over the duration of the intervention period **(B)** waist-to-length ratio **(C)** relative abdominal fat mass **(D)** leptin **(E)** fasting glycemia **(F)** fasting insulin **(G)** QUICKI **(H)** plasma lipids **(I)** White blood cell count of mice submitted to control (CTRL) or cafeteria (CAF) diet for 18 weeks. Displayed are mean ± SD **(A)** and means with individual data points **(B–I)**. QUICKI = quantitative insulin sensitivity check index, HDL, high-density lipoprotein. Statistical analysis was done using Repeated-Measure Two-Way ANOVA with Šídák’s multiple comparisons test **(A)**, unpaired t-test with two-tailed *p*-value **(B–H)** and lognormal *t*-test with two-tailed *p*-value **(I)** with *n* = 6–7 per group. For panels **(B–I)**, *p*-values were adjusted for multiple testing using the Benjamini–Hochberg false discovery rate procedure within marker families (anthropometry, metabolic markers, plasma lipids, blood counts). Statistical differences are indicated with ^*^p < 0.05, ^**^p < 0.01, ^***^p < 0.001, and ^****^p < 0.0001.

**Figure 2:**
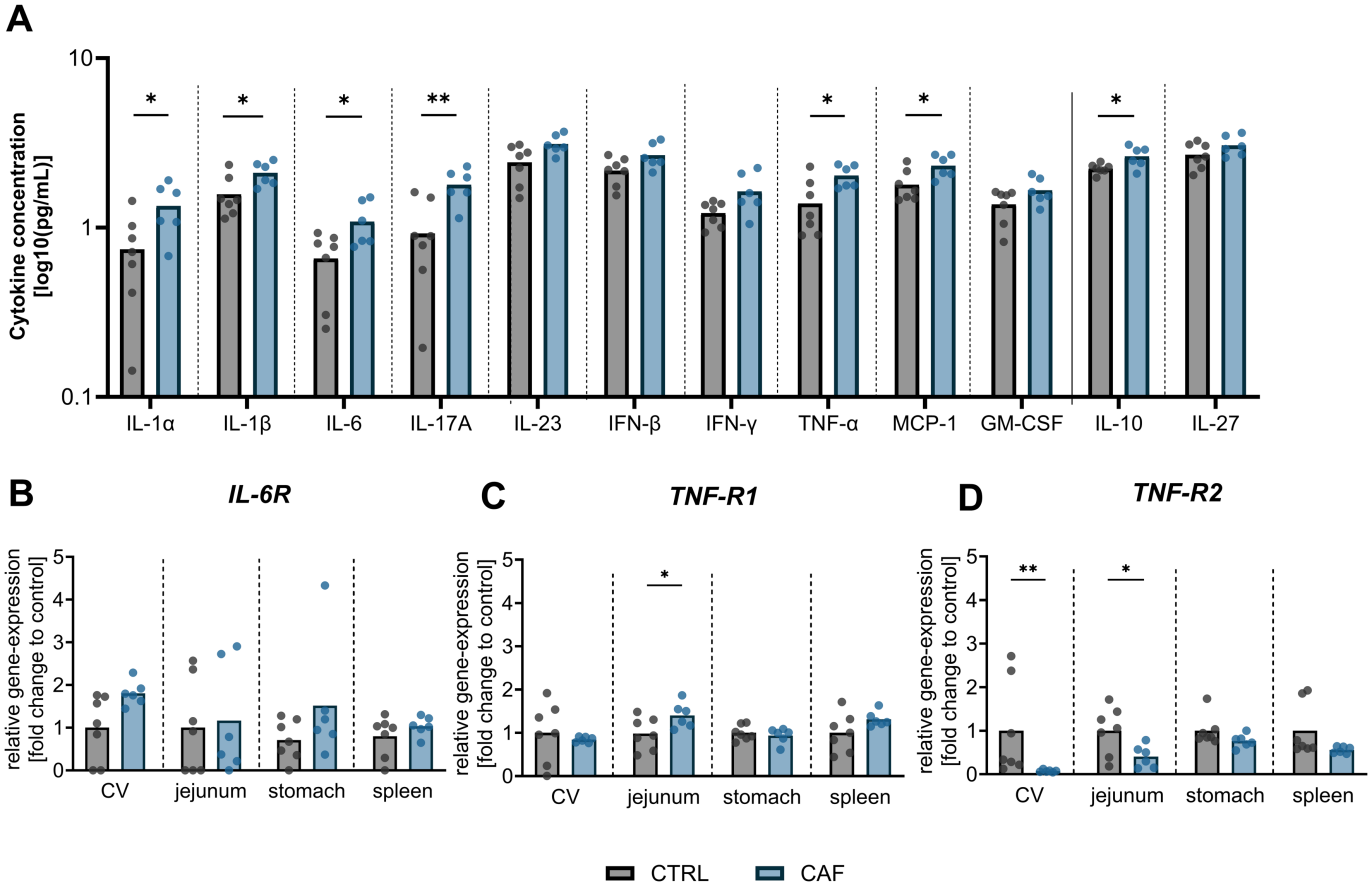
Systemic and local markers of inflammation. **(A)** Cytokine concentrations in plasma and gene-expression of **(B)** IL-6 receptor (IL-6R) **(C)** TNF-α receptor 1 (TNF-R1) and **(D)** TNF-α receptor 2 (TNF-R2) in different tissues of mice submitted to control (CTRL) or cafeteria (CAF) diet for 18 weeks. Displayed are medians with individual data points. Gene-expression values were normalized to the geometric mean of three housekeeping genes (18s, Actb, Hprt1) and are depicted as fold change to the mean control values. Statistical analysis was done using lognormal *t*-test with two-tailed *p*-value with *n* = 6–7 per group. *p*-values were adjusted for multiple testing using the Benjamini–Hochberg false discovery rate. Statistical differences are marked with ^*^p < 0.05 and **p < 0.01. IL-1α, interleukin-1 alpha; IL-1β, interleukin-1 beta; IL-6, interleukin-6; IL-17A, interleukin-17A; IL-23, interleukin-23; IFN-β, interferon-beta; IFN-γ, interferon-gamma; TNF-α, tumor necrosis factor-alpha; MCP-1, monocyte chemoattractant protein-1; GM-CSF, granulocyte-macrophage colony-stimulating factor; IL-10, interleukin-10; IL-27, interleukin-27; CV, circumvallate papillae; IL-6R, interleukin-6 receptor; TNF-R1, tumor necrosis factor-alpha receptor 1; TNF-R2, tumor necrosis factor-alpha receptor 2.

**Figure 3:**
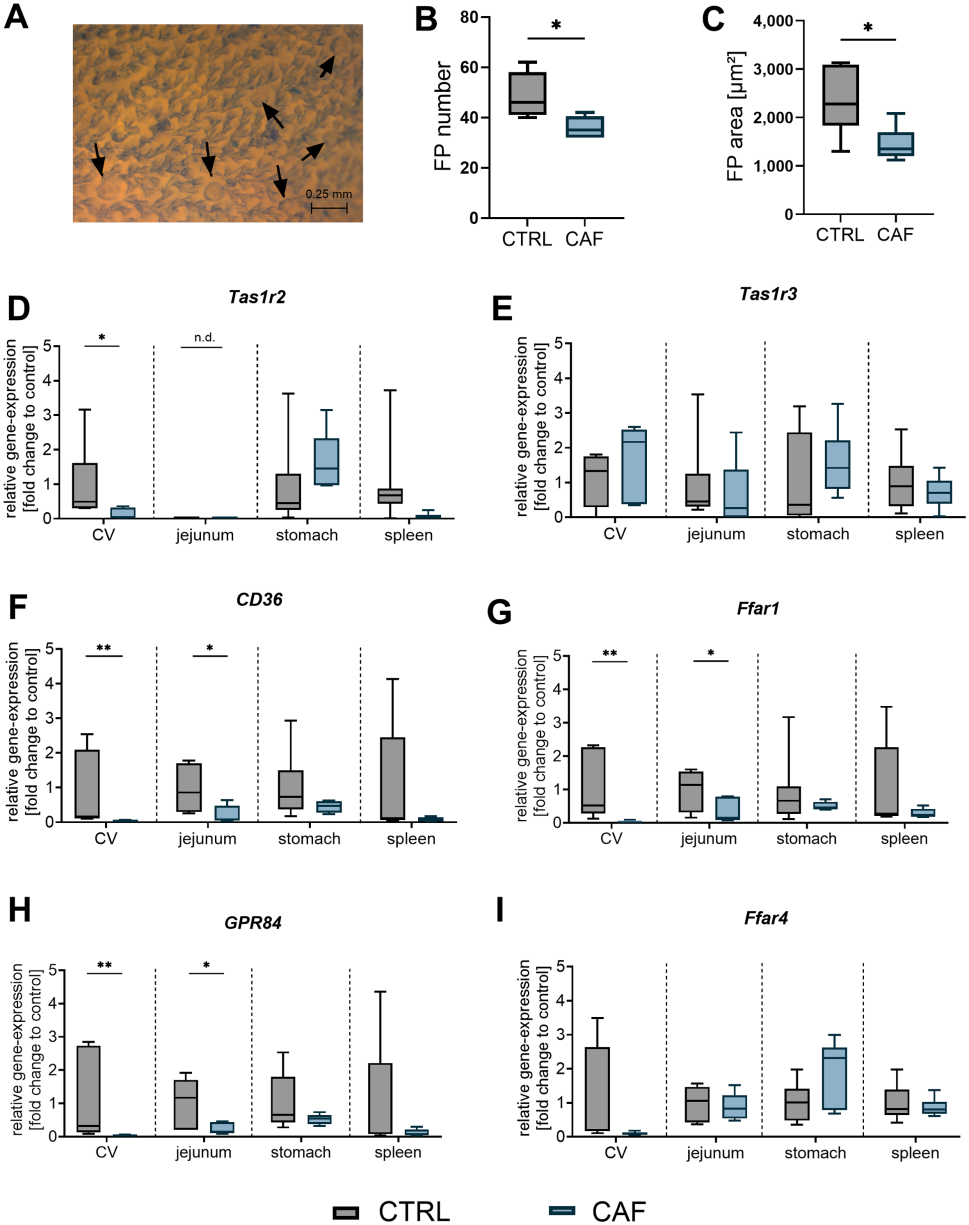
Fungiform papillae morphology and taste receptor expression. **(A)** Representative image of the anterior part of the tongue stained with methylene blue taken at 5× magnification. Fungiform papillae can be identified as light-colored circular shapes surrounded by darker blue filiform papillae and are indicated by black arrows. Fungiform Papillae (FP) **(B)** number and **(C)** area. **(D-I)** Gene-expression values of taste receptors compared throughout different tissues in mice submitted to control (CTRL) or cafeteria (CAF) diet for 18 weeks. Displayed are means with individual data points. Gene-expression values were normalized to the geometric mean of three housekeeping genes (18s, Actb, Hprt1) and are depicted as fold change to the mean control values. Statistical analysis of group comparisons was done using lognormal *t*-test with two-tailed *p*-value, with *n* = 6–7 per group. *p*-values were adjusted for multiple testing using the Benjamini–Hochberg false discovery rate. Statistical differences are indicated with ^*^p < 0.05, ^**^p < 0.01 and ^***^p < 0.001. n.d., not detectable; Tas1r2, taste receptor type 1 member 2; Tas1r3, taste receptor type 1 member 3; CD36, fatty acid translocase; Ffar1, free fatty acid receptor 1; GPR84, G-protein coupled receptor 84; Ffar4, free fatty acid receptor 4; CV, circumvallate papillae.

For statistical analysis, GraphPad Prism 10.2.3 and RStudio (*Version 2025.05.1*) were used. Normality was assessed by the Shapiro–Wilk test and by checking the corresponding QQ plots. To test for equal variance, the corresponding homoscedasticity plots in combination with a Brown-Forsythe test were examined. Group comparisons were assessed using Student’s *t*-test or lognormal *t*-test, depending on normal distribution, and two-way ANOVA with Šídák’s multiple comparison test for analyzing body weight progression. Correlations between taste markers and nutrient intake were analyzed using non-parametric Spearman Correlation. *p*-values were adjusted for multiple testing using the Benjamini–Hochberg false discovery rate. Families for FDR correction were defined based on the biological question addressed by each analysis:

- Phenotypic markers (Figure 1): correction within marker categories (anthropometry, metabolic markers, plasma lipids, blood counts)
- Plasma cytokines (Figure 2A): the full panel of 12 cytokines as one family
- Local inflammatory receptors (Figures 2B–D): within each receptor across tissues
- FP morphology (Figures 3B–C): number and area as one family
- Taste receptor expression (Figures 3D–I): within each receptor across tissues
- Dietary intake correlations (Table 1): within each marker across nutrient and energy intake variables

**Table 1:** Correlation analysis between markers for taste perception (taste receptors in CV and morphological aspects of fungiform papillae) and carbohydrate, fat and caloric intake.

Taste marker	Protein intake [g]	Carbohydrate intake [g]	Fat intake [g]	Caloric intake [kcal]
Tas1r2	*r* = −0.07, n.s.	*r* = −0.60, *p* < 0.05	*r* = −0.62, *p* < 0.05	*r* = −0.64, *p* < 0.05
Tas1r3	*r* = −0.04, n.s.	*r* = 0.22, n.s.	*r* = 0.24, n.s.	*r* = 0.22, n.s.
CD36	*r* = 0.11, n.s.	*r* = −0.48, n.s.	*r* = −0.75, *p* < 0.01	*r* = −0.77, *p* < 0.01
Ffar1	*r* = 0.15, n.s.	*r* = −0.79, *p* < 0.01	*r* = −0.80, *p* < 0.01	*r* = −0.78, *p* < 0.01
GPR84	*r* = 0.09, n.s.	*r* = −0.47, n.s.	*r* = −0.80, *p* < 0.01	*r* = −0.80, *p* < 0.01
Ffar4	*r* = 0.12, n.s.	*r* = −0.43, n.s.	*r* = −0.79, *p* < 0.01	*r* = −0.80, *p* < 0.01
FP number	*r* = 0.20, n.s.	*r* = −0.40, n.s.	*r* = −0.54, n.s.	*r* = −0.53, n.s.
FP area	*r* = −0.28, n.s.	*r* = −0.53, n.s.	*r* = −0.54, n.s.	*r* = −0.58, n.s.

Statistical analysis was done using non-parametric Spearman Correlation, *n* = 13. *p*-values were adjusted for multiple testing using the Benjamini–Hochberg false discovery rate. Statistical differences are indicated with ^*^p < 0.05, ^**^p < 0.01 and n.s., not significant; Tas1r2, taste receptor type 1 member 2; Tas1r3, taste receptor type 1 member 3; CD36, fatty acid translocase; Ffar1, free fatty acid receptor 1; GPR84, G-protein coupled receptor 84; Ffar4, free fatty acid receptor 4; FP, fungiform papillae.

## 3 Results

### 3.1 An 18-week cafeteria diet induced obesity and metabolic dysfunction

Mice fed with standard chow consumed 35.5% of carbohydrates, 26.4% of fat, 34.8% of protein, and 3.3% of fiber, with fixed caloric intake from the given macronutrients. Based on voluntary selection, mice on the CAF diet consumed 39.9% of carbohydrates, 36.6% of fat, 21.5% of protein, and 2% of fiber, corresponding to 27.6% of carbohydrates, 56.9% of fat, 14.9% of protein, and 0.7% of fiber of caloric intake from the given macronutrients. From week 5 on, CAF-fed mice showed continuous weight gain, while CTRL mice maintained stable body weights (Figure 1A). At the end of the study, CTRL mice weighed on average 24.3 ± 0.6 g, whereas CAF mice averaged 46.7 ± 2.9 g, corresponding to a 92.1 ± 2.4% higher body weight in the CAF group (*p* < 0.0001, Figure 1A). CAF mice also exhibited central obesity, as reflected by larger waist-to-length ratio (*p* < 0.0001, Figure 1B), and relative abdominal fat mass compared to CTRLs (*p* < 0.0001, Figure 1C). Correspondingly, leptin levels were increased in CAF-fed mice (*p* < 0.001, Figure 1D). CAF mice showed metabolic syndrome-like pathology, including a higher fasting glycemia (by 57.8 ± 5.8%, *p* < 0.0001, Figure 1E), and 12-fold higher fasting insulinemia compared to controls (*p* < 0.0001, Figure 1F), resulting in 36.54 ± 7.47% lower quantitative insulin sensitivity check index (QUICKI, *p* < 0.001, Figure 1G). Additionally, CAF mice showed higher total cholesterol and non-HDL-C concentrations compared to lean counterparts (all: *p* < 0.05, Figure 1H). The plasma TAG concentrations did not differ between the two groups. Leukocyte and neutrophil numbers were higher in CAF mice compared to lean ones (*p* < 0.01); however, lymphocyte counts did not differ (Figure 1I).

Collectively, these findings confirm that the CAF diet robustly induced obesity and cardiometabolic dysfunction, accompanied by signs of systemic inflammation, as indicated by elevated leukocyte and neutrophil counts.

### 3.2 Inflammatory response to diet-induced obesity occurs systemically and locally

The analysis of selected cytokines also confirmed systemic inflammation as shown by significantly higher IL-1α, IL-1β, IL-6, IL-17A, TNF-α, MCP-1, and IL-10 levels in CAF mice compared to controls (Figure 2A). Local inflammatory responses were assessed via gene expression of receptors for IL-6 (*IL-6R*) and TNF-α (*TNF-R1*, *TNF-R2*) across CV, lower jejunum, stomach, and spleen. In CAF mice *TNF-R1* was increased only in lower jejunum (*p* < 0.05, Figure 2C), while *TNF-R2* was downregulated by 87% in CV (geometric mean: 0.072 vs. 0.555, ratio 0.13, 95% CI: 0.04–0.40, *p* < 0.01) and by 59% in lower jejunum (geometric mean: 0.334 vs. 0.808, ratio 0.41, 95% CI: 0.16–0.99, *p* < 0.05, Figure 2D), with trends toward downregulation in stomach and spleen (*p* < 0.1).

### 3.3 Fungiform papillae morphology and CV taste receptor expression are decreased in diet-induced obesity

The total number and size of FP (Figure 3A) was assessed as morphological markers, and taste receptor gene expression in CV as molecular markers of the taste system. FP number and area differed between groups at the end of the dietary intervention. FP number was decreased by 24% (geometric mean: 35.80 vs. 47.37, ratio 0.76, 95% CI of ratio: 0.63–0.91, *p* < 0.05, Figure 3B) and area by 36% (geometric mean: 1,418 vs. 2,223 μm^2^, ratio 0.64, 95% CI of ratio: 0.46–0.89, *p* < 0.05, Figure 3C) in the CAF compared to the control group, with both parameters correlating significantly with each other (*r* = 0.77, *p* < 0.01).

Taste receptor gene expression analysis revealed *Tas1r2* expression was reduced by 85% in CV tissue of CAF mice compared to controls (geometric mean: 0.099 vs. 0.676, ratio 0.15, 95% CI: 0.03–0.79, *p* < 0.05, Figure 3D). In contrast to *Tas1r2*, *Tas1r3* expression showed no significant differences across the tested tissues between the diet groups (Figure 3E). Decreased gene-expression levels of *CD36*, *Ffar1*, and *GPR84* were detected in both CV and lower jejunum of CAF mice (Figures 3F–H), while *Ffar4* remained unchanged Figure 3I).

Housekeeping gene-normalized gene-expression values for all measured receptors across the four tissues are provided in Supplementary Table S7, allowing assessment of basal receptor expression levels alongside the diet-induced changes reported here.

### 3.4 Gustatory taste receptor gene expression correlates with nutrient intake

To evaluate the relationship of gustatory sweet and fatty taste receptor gene expression with dietary intake, we correlated CV sweet and fatty acid receptor expression with corresponding nutrient and energy intake (Table 1). *Tas1r2*, but not *Tas1r3*, correlated negatively with carbohydrate (*r* = −0.60, *p* < 0.05), fat intake (*r* = −0.62, *p* < 0.05), and total caloric intake (*r* = −0.64, *p* < 0.05). All fatty acid receptors correlated with fat and caloric intake, while *Ffar1* additionally correlated with carbohydrate intake (*r* = −0.79, *p* < 0.01). Beyond gene expression, we examined how morphological characteristics of FP relate to dietary intake patterns. However, neither FP number nor FP area showed significant correlations with macronutrient or energy intake.

### 3.5 Correlation network analysis reveals tissue-specific taste-inflammatory associations

**Figure 4:**
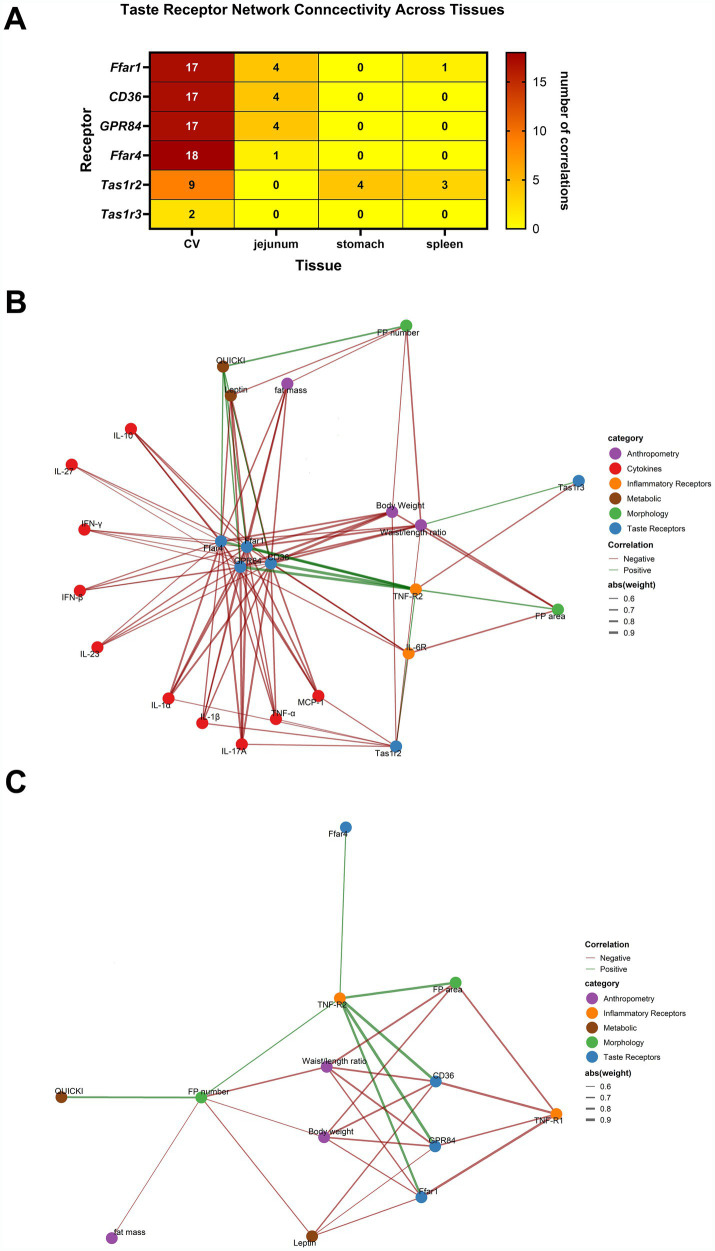
Taste marker connectivity across tissues. **(A)** Heatmap depicting the number of network connections for analyzed taste receptors throughout different tissues. **(B)** Network analysis of taste markers (taste receptors and fungiform papillae). Depicted receptors for taste (Tas1r2, Tas1r3, CD36, Ffar1, GPR84, Ffar4) inflammation (IL-6R, TNF-R1, TNF-R2) were analyzed in circumvallate papillae (CV) and **(C)** lower jejunum. The network shows correlations with │*r*│ > 0.5 and FDR-adjusted *p* < 0.05, positive correlations are displayed in green, negative correlations in red, line thickness indicates correlation strength. Pairwise correlations between all variables were calculated using Spearman’s rank correlation coefficient and *p*-values were adjusted for multiple testing using the Benjamini–Hochberg false discovery rate. Tas1r2, taste receptor type 1 member 2; Tas1r3, taste receptor type 1 member 3; CD36, fatty acid translocase; Ffar1, free fatty acid receptor 1; GPR84, G-protein coupled receptor 84; Ffar4, free fatty acid receptor 4; CV, circumvallate papillae.

To assess how sweet and fatty acid receptors and fungiform papillae characteristics are associated with inflammatory, cardiometabolic, and morphometric markers, and whether patterns vary between tissues, we performed organ-specific network analysis based on correlation analyses (│*r*│ > 0.5, FDR adjusted *p* < 0.05). A complete edge-level summary including Spearman *r*, raw and FDR-adjusted *p*-values, and pairwise sample sizes is provided in Supplementary Tables S3–S6. Permutation testing confirmed that the observed network densities exceeded chance expectation in all four tissues (empirical *p* < 0.001; null distributions maximum 2–3 edges across 1,000 permutations vs. 4–66 observed cross-domain edges depending on tissue). Network connectivity differed across tissues, with CV tissue exhibiting the highest number of significant correlations (Figure 4A).

Figure 4B visualizes the correlation network in CV tissue. Fatty acid receptors exhibited similar connectivity patterns, with negative correlations with systemic inflammatory markers, morphometric parameters (body weight, relative abdominal fat mass, waist/length ratio, and leptin), but positive correlations with QUICKI and local *TNF-R2* expression in CV. *Tas1r2* correlated positively with *TNF-R2* and showed associations with multiple cytokines, but less connectivity to morphometric and cardiometabolic markers. In addition, *Tas1r2* was negatively associated with waist/length ratio and body weight, but there was no connection to the relative abdominal fat mass. *Tas1r2* also correlated negatively with local *IL-6R* expression. *Tas1r3* displayed opposite correlation patterns.

With respect to the morphological markers, neither FP area nor FP number correlated with circulating cytokine levels. FP area showed strong associations with morphometric markers (body weight, waist/length ratio) and correlated with *TNF-R2* and *IL-6R* expression levels in the CV. In contrast, FP number was more strongly associated with cardiometabolic parameters (QUICKI, leptin and relative abdominal fat mass).

The jejunum correlation network revealed different patterns compared to CV, with reduced overall connectivity (Figure 4C). Systemic inflammatory parameters showed no correlations with sweet and fatty acid receptors. However, taste receptor-inflammatory relationships persisted at the local level. All fatty acid receptors showed positive correlations with jejunal *TNF-R2* expression, while *CD36*, *Ffar1* and *GPR84* were additionally inversely associated with jejunal *TNF-R1*. In addition, these fatty acid receptors correlated with body weight, waist/length ratio, and leptin. *Tas1r2* and *Tas1r3* showed no correlations, contrasting with CV tissue. Stomach (Supplementary Figure S8) and spleen (Supplementary Figure S9) showed overall sparse interaction patterns. With the exception of *Ffar1* in the spleen, fatty acid receptors exhibit no correlations in either tissue, contrary to CV and lower jejunum. Within the stomach network, *Tas1r2* showed positive correlations to IL-6, QUICKI, and relative abdominal fat mass. Splenic *Tas1r2,* showed no connectivity with inflammatory receptors. Instead, decreased *Tas1r2* expression was associated with elevated relative abdominal fat mass, body weight, waist/length ratio, and leptin.

## 4 Discussion

We used a CAF diet-induced obesity model with related metabolic complications in mice to investigate whether taste receptor-inflammation connections occur tissue-independently, hypothesizing that obesity-associated inflammatory markers would be associated with altered sweet and fatty acid receptor gene expression across both gustatory and extra-oral tissues. The CAF diet induced general and central obesity accompanied by the manifestation of classical cardiometabolic risk factors (insulin resistance and dyslipidemia), systemic low-grade inflammation, and alterations in taste system markers. Our findings align with previous reports showing that the CAF diet induces more pronounced metabolic dysfunction and systemic inflammation compared to HFD (32). At the local level, inflammatory receptor expressions showed tissue-specific patterns. *TNF-R1* was upregulated in the lower jejunum of CAF mice, while *TNF-R2* was downregulated in both CV and lower jejunum compared to control mice. TNF-R1 primarily mediates proinflammatory responses (33, 34), while TNF-R2 is associated with anti-inflammatory effects (35). The concurrent upregulation of *TNF-R1* and downregulation of *TNF-R2* is therefore consistent with enhanced local proinflammatory signaling and a diminished anti-inflammatory response, in line with the inflammatory phenotype detected in CAF mice.

Beyond inflammatory changes, we detected alterations in two anatomically distinct components of the taste system: morphological changes in fungiform papillae (FP) on the anterior tongue, and molecular changes in circumvallate papillae (CV) on the posterior tongue. This anatomical separation was a consequence of the limited tissue available, as the small size of the CV required the entire tissue for RNA extraction, leaving no material for histological analysis. As a result, FP morphology and CV gene expression cannot be directly integrated within the same anatomical structure, and findings from each should be interpreted as reflecting different parts of the gustatory system. The number and area of FP were reduced, aligning with previous studies demonstrating connections between obesity, diet, and FP morphology. Kaufman et al., for instance, showed that diet-induced obese mice had reduced FP density, and in humans, FP density correlated negatively with neck circumference, a marker for adiposity (36). Similarly, FP density has been shown to correlate negatively with BMI (37), body weight, and body fat (38), and also the number of FP was associated with body weight and white adipose tissue mass (17). A previous study from our group additionally showed that addition of sugar-sweetened beverages to a Western-style diet reduced FP chemosensory surface area without producing differences in body weight or cardiometabolic markers between groups (18), indicating that FP morphology can change in response to dietary composition independently of body weight gain. The FP changes observed in the present study could therefore reflect dietary effects rather than obesity-associated pathology, although the cross-sectional design precludes determining the temporal relationship between dietary exposure, body weight changes, and FP morphology.

At the molecular level, *Tas1r2* expression was lower in CV tissue of mice fed the sugar-rich CAF diet. This aligns with findings by Shahbandi et al. (39), who demonstrated reduced *Tas1r2* expression in CV taste buds following sub-acute saccharin exposure. However, *Tas1r3* expression showed no significant alterations between the diet groups in any of the tested tissues, which contrasts with other studies reporting a downregulation of *Tas1r3* as a response to diet-induced obesity (19, 22, 40, 41). While this differential regulation of the two sweet taste receptor subunits may appear unexpected, it could be explained by their distinct functional roles. Unlike Tas1r2, which is specific to sweet taste perception, Tas1r3 forms heterodimers with Tas1r2 (sweet receptor) (42) and Tas1r1 (umami receptor) (43). Therefore, Tas1r3 is not selective for sweet taste signaling. Thus, the differential regulation of *Tas1r2* and *Tas1r3* could indicate that the subunits of the sweet taste receptor are subject to distinct regulatory mechanisms, although confirming this would require dedicated mechanistic studies.

The fatty acid receptors *CD36*, *Ffar1* and *GPR84* were reduced in the CV tissue of CAF mice. For *CD36*, similar gustatory downregulation following HFD has been reported previously (44). *CD36*, *Ffar1*, and *GPR84* were also downregulated in the lower jejunum of CAF mice. Given that CAF mice exhibit obesity and elevated blood lipids, the intestinal downregulation of these receptors could represent a metabolic adaptation to reduce further fat uptake in response to already sufficient lipid availability, although confirming this interpretation would require functional readouts beyond gene expression, and the cross-sectional design precludes determining whether receptor changes precede, parallel, or follow the obesity and lipid alterations observed. In contrast, *Ffar4* expression remained unchanged in both CV and lower jejunum. This could be due to Ffar4’s preferential responsiveness to omega-3 fatty acids (45), which were not enriched in the CAF diet, whereas CD36, Ffar1, and GPR84 respond to the saturated and unsaturated fats abundant in the CAF diet.

*Tas1r2* expression in CV was negatively associated with carbohydrate, fat, and total caloric intake. All fatty acid receptors showed strong negative correlations with fat and caloric intake, consistent with previous results showing associations between reduced fatty acid taste receptor expressions and increased preference and consumption of fat-rich foods (46, 47). Although FP number and area were significantly reduced in CAF mice, neither correlated significantly with individual dietary intake measures. Collectively, these correlations suggest that the expression of sweet (*Tas1r2*) and fatty acid (*CD36*, *Ffar1*, *GPR84*, *Ffar4*) receptors in CV is more closely linked to nutrient and energy intake than FP morphology.

Network analyses of correlations between sweet and fatty acid receptors, inflammatory, morphometric, and cardiometabolic parameters across the four tissues studied indicated tissue-specific patterns of correlation rather than the hypothesized universal pattern across gustatory and extra-oral tissues. Given the small group sizes (*n* = 6–7 per group) and the resulting limited statistical power, these network analyses are regarded as exploratory with the aim of identifying potential correlations. CV tissue exhibited the highest network connectivity and was the only tissue in which sweet and fatty acid receptor expression correlated with systemic inflammatory markers (IL-1α, IL-1β, IL-10, IL-17A, IFN-β, TNF-α, MCP-1), alongside local inflammatory receptors (*IL-6R*, *TNF-R2*), morphometric, and cardiometabolic parameters. The relationships between taste receptors and inflammatory markers are consistent with prior work by Archer et al., who reported upregulation of inflammatory pathway gene expression alongside reduced expression of taste signaling genes in the FP of obese individuals (21), but also align with documented associations between inflammation and taste alterations across various diseases (48–50).

FP morphology (number and area) showed connectivity patterns distinct from those observed for CV gene expression. As discussed above, these two readouts come from different anatomical structures rather than from different facets of a single tissue, and the contrast between them should be interpreted with this anatomical separation in mind. The FP area showed strong associations with morphometric markers, supporting previous findings (36, 37, 51, 52). Notably, neither FP measure correlated with systemic cytokines, in contrast to associations reported between taste bud density and inflammation (20).

Interestingly, within the CV network, *Tas1r2* expression correlated negatively with body weight and waist/length ratio but not with relative abdominal fat mass, whereas fatty acid receptors correlated with both. A partially parallel pattern was shown in an observational human study from our working group, where sucrose recognition thresholds and IL-6 levels clustered separately from body composition in a principal component analysis, while FP morphology was associated with fat mass (52). Despite substantial differences in the respective contexts, both studies suggest that sweet taste markers may be associated with inflammatory parameters in a manner partly distinct from associations with body composition. In contrast, FP morphology correlates with body composition but not inflammation. Together, these patterns are consistent with distinct regulatory influences on CV *Tas1r2* expression and FP morphology, although confirming this would require longitudinal and mechanistic studies.

In the lower jejunum, sweet and fatty acid receptors showed reduced connectivity compared to CV, with the surviving correlations involving primarily fatty acid receptors. In contrast to the gustatory tissue, systemic inflammatory correlations were absent. However, taste receptor-inflammatory relationships persisted on a local level. The fatty acid receptors showed positive correlations with jejunal *TNF-R2* expression, while *Ffar1*, *GPR84,* and *CD36* were additionally negatively associated with jejunal *TNF-R1.* In addition, these fatty acid receptors correlated with body weight, waist/length ratio, and leptin. The absence of *Tas1r2*/*Tas1r3* correlations in the lower jejunum, contrasting sharply with CV, is consistent with this tissue-specific pattern of taste receptor regulation, although mechanistic differences cannot be inferred from correlation data alone.

In contrast to the CV and lower jejunum, the stomach and spleen showed sparse connectivity networks, with fatty acid receptors nearly absent from correlation networks in both tissues. Within the stomach network, *Tas1r2* showed a negative correlation to QUICKI, contrasting with CV tissue. However, given that gastric *Tas1r2* expression did not differ significantly between groups, the interpretation of these correlations is therefore limited. Despite the spleen’s central role in immune regulation, splenic *Tas1r2* showed no connectivity with cytokines or inflammatory receptors but was negatively correlated with the morphometric markers body weight, waist/length ratio, body fat, as well as with leptin. As with gastric *Tas1r2*, splenic *Tas1r2* expression did not differ significantly between groups after FDR correction, limiting the interpretation of these correlations.

These exploratory network analyses indicated that correlations between sweet and fatty acid receptor expression and inflammatory markers were concentrated in CV tissue, which exhibited the highest network connectivity and was the only tissue in which these receptors correlated with systemic inflammatory markers. In contrast, the lower jejunum showed reduced connectivity with loss of systemic inflammatory associations, while the stomach and spleen demonstrated sparse networks with fatty acid receptors absent from correlation networks. This tissue-specific pattern does not support our initial hypothesis of universal taste-inflammation associations across gustatory and extra-oral tissues.

There are methodological limitations that should be considered when interpreting these findings. While gustatory *Tas1r2* and fatty acid receptor expression correlated with nutritional intake, supporting relevance, taste sensitivity testing would have strengthened these interpretations. Furthermore, as the present analyses are based on mRNA expression, they do not capture protein abundance, receptor localization, or functional taste signaling. Consequently, complementary approaches such as immunohistochemistry, western blot or targeted proteomics, taste nerve recordings, brief-access lick testing, or measurement of postprandial gut hormone responses (GLP-1, CCK, PYY) would be needed to link the observed expression changes to receptor function and downstream physiological effects. Such protein-level validation was not feasible in this study because the small size of the CV required the entire tissue for RNA extraction. The sample size, determined *a priori* to detect differences in body weight, limited statistical power for the molecular and network analyses, which should accordingly be regarded as exploratory. In addition, the cafeteria diet differs from standard chow in multiple respects—including macronutrient composition, palatability, texture, sweetness, and total caloric intake—features that together promote the hyperphagia and obesity that characterize this model. As a consequence of this multifactorial nature, the present design cannot fully separate the contributions of diet composition, hyperphagia, obesity, and systemic inflammation to the observed taste receptor changes. Therefore, designs incorporating pair-fed, high-fat-only, or calorie-matched comparison groups could help disentangle these factors. However, prior findings from our group indicate that FP morphological changes can occur independently of body weight gain (18), and a study using captopril to prevent high-fat-diet-induced weight gain found reduced expression of taste signaling components (gustducin, PLCβ2) regardless of whether obesity developed (53), suggesting that not all taste system alterations under obesogenic dietary conditions are obesity-driven. Lastly, only female mice were studied; given documented sex-related differences in taste signaling (54), immune responses (55, 56) and diet-induced obesity phenotypes (57), the present findings should not be generalized to males without further investigation. Future investigations examining additional metabolism-relevant tissues, such as pancreas and muscle tissue, could further elucidate tissue-specific taste receptor connections. Despite these limitations, this study provides initial evidence suggesting that taste receptor-inflammation interactions may exhibit pronounced tissue-specificity rather than universal patterns, with systemic inflammatory associations predominantly occurring in gustatory tissue. This emphasizes the importance of anatomical context when studying taste receptor function and regulation.

## 5 Conclusion and outlook

Given their established roles in metabolic regulation, we here examined whether extra-oral sweet and fatty acid receptors exhibit inflammation-associated expression patterns similar to gustatory tissue after induction of metabolic dysfunction with a CAF diet. Our findings revealed pronounced tissue-specificity in taste receptor-inflammation associations: taste receptor-systemic inflammation networks were predominantly localized to CV tissue, while extra-oral sweet and fatty acid receptors in lower jejunum, stomach, and spleen showed limited or absent systemic inflammatory associations. These distinct correlation patterns suggest that gustatory expression of sweet and fatty acid receptors is more strongly associated with systemic inflammatory changes in the context of CAF-diet-induced obesity. Gustatory and extra-oral sweet and fatty acid receptors may thus operate under distinct regulatory frameworks, emphasizing the importance of anatomical context when studying taste receptors.

The selective association of gustatory tissue with obesity-associated inflammatory signals may impact food intake regulation. Reduced expression of sweet and fatty acid receptors in the CV could, in principle, impair the detection of these stimuli and the satiety signals associated with them, contributing to hyperphagia (58),—a hypothesis that requires direct functional testing to verify. Our findings are consistent with a previously proposed self-reinforcing cycle: diminished taste signaling promotes overconsumption, which further drives weight gain and inflammation, which in turn may exacerbate receptor downregulation (14). However, whether taste receptor changes precede, parallel, or follow the progression of obesity cannot be determined from the present cross-sectional design. Beyond the oral cavity, the observed downregulation of fatty acid receptors in the lower jejunum in the absence of strong inflammatory network connectivity raises the question of key drivers of this regulation. Alternative mechanisms, such as shifts in gut microbiota composition, thus need to be addressed in future studies. Since intestinal fatty acid receptors are functionally coupled to satiety signaling, whether their reduced expression translates into impaired postprandial hormonal responses and contributes to overconsumption through mechanisms similar to those proposed for gustatory tissue remains an open question requiring direct experimental investigation.

## Data Availability

The gene expression dataset generated and analyzed in this study is available on Zenodo under https://doi.org/10.5281/zenodo.20074686.

## Glossary

Actb: beta-actin
CAF: cafeteria diet
CCK: cholecystokinin
CD36: fatty acid translocase
CTRL: control
CV: circumvallate papillae
EDTA: ethylenediaminetetraacetic acid
Ffar1: free fatty acid receptor 1
Ffar4: free fatty acid receptor 4
FP: fungiform papillae
GM-CSF: granulocyte-macrophage colony-stimulating factor
GPR84: G-protein-coupled receptor 84
HDL: high-density lipoprotein
HFD: high fat diet
HPRT1: hypoxanthine phosphoribosyltransferase 1
IFN-β: interferon-beta
IFN-γ: interferon-gamma
IL-1α: interleukin-1 alpha
IL-1β: interleukin-1 beta
IL-6: interleukin-6
IL-6R: interleukin-6 receptor
IL-10: interleukin-10
IL-17A: interleukin-17A
IL-23: interleukin-23
IL-27: interleukin-27
MCP-1: monocyte chemoattractant protein-1
PBS: phosphate-buffered saline
PYY: peptide YY
QUICKI: quantitative insulin sensitivity check index
SGLT1: sodium/glucose cotransporter 1
TAG: triacylglycerols
TAS1R2/3: taste 1 receptor member 1/2/3 [human]
Tas1r1/2/3: taste receptor, type 1, member 1/2/3 [*Mus musculus*]
TNF-α: tumor necrosis factor-alpha
TNF-R1: tumor necrosis factor-alpha receptor 1
TNF-R2: tumor necrosis factor-alpha receptor 2
WBC: white blood cell count
