# Supplementary material for: Taste receptor downregulation in diet-induced obesity reveals circumvallate papillae-specific taste-inflammatory networks
Source: Front Nutr. 2026 Jun 24;13:1857562. doi: 10.3389/fnut.2026.1857562 (PMC13342050; doi:10.3389/fnut.2026.1857562)
Supplement: Supplementary file 1 [file Supplementary_file_1.DOCX]

Supplementary Material

***Table S1:*** *Composition of the Cafeteria diet. Caloric and macronutrient content are presented as given by the manufacturer per 100g.*

|  | **kcal** | **Fat [%]** | **Carbohydrate [%]** | **Protein [%]** | **Fibre [%]** | **Salt [%]** |
| --- | --- | --- | --- | --- | --- | --- |
| **MENU 1** |  |  |  |  |  |  |
|  |  |  |  |  |  |  |
| **Cake** | 404 | 24 | 39 | 6.1 | 2.1 | 1 |
| **Cheese** | 458 | 40 | 2 | 22 |  | 3.3 |
| **Candies** | 570 | 37 | 52 | 5 | 4.3 | 0.2 |
| **Salty sticks** | 452 | 17 | 64 | 9.4 | 2.9 | 2.3 |
| **MENU 2** |  |  |  |  |  |  |
|  |  |  |  |  |  |  |
| **Biscuits** | 400 | 19 | 52 | 5.7 | 3.3 | 1 |
| **Salami** | 352 | 28 | 0 | 25 |  | 1.3 |
| **Cookies** | 474 | 19 | 68 | 5.4 | 2.7 | 0.7 |
| **Potato chips** | 540 | 35 | 48.4 | 5.5 | 4.4 | 1.4 |
| **MENU 3** |  |  |  |  |  |  |
|  |  |  |  |  |  |  |
| **Cake** | 473 | 29 | 48 | 4.8 | 1.7 | 0.3 |
| **Salami** | 458 | 40 | 2 | 22 |  | 3.3 |
| **Candies** | 373 | 3.7 | 80 | 4.7 |  | 0.1 |
| **Salty sticks** | 382 | 3.8 | 72 | 13 | 4 | 4 |
| **MENU 4** |  |  |  |  |  |  |
|  |  |  |  |  |  |  |
| **Biscuits** | 453 | 28 | 43 | 6 |  | 0.5 |
| **Cheese** | 352 | 28 | 0 | 25 |  | 1.3 |
| **Cookies** | 494 | 23.9 | 64.6 | 4.5 | 2 | 0.1 |
| **Potato chips** | 483 | 22.5 | 60.8 | 6.4 |  | 1.2 |

***Table S2:*** *Primer Sequences.*

| **Gene Name** | **Forward Primer** | **Reverse Primer** | **Concentration for PCR** |
| --- | --- | --- | --- |
| *Actb* | CCCTGTGCTGCTCACC | GCACGATTTCCCTCTCAG | 100 nM |
| *18S* | CGGCTACCACATCCAAGGAA | GCTGGAATTACCGCGGCT | 100 nM |
| *Hprt1* | GACCAGTCAACAGGGGACAT | CCTGACCAAGGAAAGCAAAG | 100 nM |
| *Tas1r2* | AAGCATCGCCTCCTACTCC | GGCTGGCAACTCTTAGAACAC | 300 nM |
| *Tas1r3* | GAAGCATCCAGATGACTTCA | GGGAACAGAAGGACACTGAG | 300 nM |
| *CD36* | TAGTAGGCGTGGGTCTGAAG | GCTTCAGGGAGACTGTTGAA | 100 nM |
| *Ffar1* | ATCCGAGGCGCAGTGTCCCA | AGCCTCCGCCTGCGTAGAGG | 100 nM |
| *GPR84* | AGGTGACCCGTATGTGCTTC | GTTCATGGCTGCATAGAGCA | 100 nM |
| *Ffar4* | GTGACTTTGAACTTCCTGGTGCC | CAGAGTATGCCAAGCTCAGCGT | 100 nM |
| *IL-6R* | GCCGGATCCACCTGCCAACCTT | GGGCCACCGGGAGCAGCAACAC | 100 nM |
| *TNF-R1* | CCGGGAGAAGAGGGATAGCTT | TCGGACAGTCACTCACCAAGT | 100 nM |
| *TNF-R2* | ACACCCTACAAACCGGAACC | AGCCTTCCTGTCATAGTATTCCT | 100 nM |

*Table S3: Edge Table CV.*

| **Anchor (taste/FP)** | **Partner** | **Partner Category** | **Spearman r** | **Raw**  **p-value** | **FDR-adjusted p-value** | **n** | **In network (FDR<0.05, \|r\|>0.5)** |
| --- | --- | --- | --- | --- | --- | --- | --- |
| CD36 | TNF-R2 | receptor | 0.984 | <0.0001 | <0.0001 | 13 | Yes |
| Ffar1 | TNF-R2 | receptor | 0.973 | <0.0001 | <0.0001 | 13 | Yes |
| GPR84 | TNF-R2 | receptor | 0.929 | <0.0001 | <0.0001 | 13 | Yes |
| CD36 | Body Weight | metabolic | -0.912 | <0.0001 | <0.0001 | 13 | Yes |
| Ffar4 | TNF-R2 | receptor | 0.89 | 0.000135 | 0.001 | 13 | Yes |
| Ffar1 | IL-17A | cytokine | -0.846 | <0.0001 | 0.002 | 13 | Yes |
| Ffar1 | Body Weight | metabolic | -0.863 | 0.001 | 0.003 | 13 | Yes |
| GPR84 | IL-17A | cytokine | -0.857 | 0.001 | 0.003 | 13 | Yes |
| GPR84 | Body Weight | metabolic | -0.841 | 0.000319 | 0.004 | 13 | Yes |
| CD36 | IL-17A | cytokine | -0.802 | 0.001 | 0.005 | 13 | Yes |
| Ffar1 | Leptin | metabolic | -0.808 | 0.002 | 0.007 | 13 | Yes |
| Ffar1 | IL-1a | cytokine | -0.78 | 0.002 | 0.007 | 13 | Yes |
| CD36 | Waist/length ratio | metabolic | -0.786 | 0.002 | 0.008 | 13 | Yes |
| CD36 | IL-1a | cytokine | -0.78 | 0.002 | 0.008 | 13 | Yes |
| CD36 | Leptin | metabolic | -0.769 | 0.003 | 0.008 | 13 | Yes |
| Ffar4 | IL-17A | cytokine | -0.808 | 0.002 | 0.008 | 13 | Yes |
| Ffar4 | IL-1a | cytokine | -0.797 | 0.002 | 0.008 | 13 | Yes |
| Ffar1 | Waist/length ratio | metabolic | -0.786 | 0.003 | 0.009 | 13 | Yes |
| FP area | Waist/length ratio | metabolic | -0.786 | 0.00145 | 0.01 | 13 | Yes |
| GPR84 | IL-1a | cytokine | -0.78 | 0.003 | 0.011 | 13 | Yes |
| FP number | QUICKI | metabolic | 0.761 | 0.0025 | 0.013 | 13 | Yes |
| GPR84 | Waist/length ratio | metabolic | -0.758 | 0.00267 | 0.014 | 13 | Yes |
| CD36 | Relative abdominal fat mass | metabolic | -0.736 | 0.006 | 0.014 | 13 | Yes |
| CD36 | MCP-1 | cytokine | -0.736 | 0.005 | 0.014 | 13 | Yes |
| Ffar1 | Relative abdominal fat mass | metabolic | -0.731 | 0.005 | 0.014 | 13 | Yes |
| Ffar1 | MCP-1 | cytokine | -0.725 | 0.005 | 0.014 | 13 | Yes |
| Ffar4 | Body Weight | metabolic | -0.78 | 0.004 | 0.015 | 13 | Yes |
| Ffar4 | IL-10 | cytokine | -0.72 | 0.005 | 0.015 | 13 | Yes |
| CD36 | TNF-a | cytokine | -0.725 | 0.007 | 0.016 | 13 | Yes |
| Ffar1 | QUICKI | metabolic | 0.687 | 0.007 | 0.016 | 13 | Yes |
| GPR84 | MCP-1 | cytokine | -0.742 | 0.006 | 0.016 | 13 | Yes |
| GPR84 | Relative abdominal fat mass | metabolic | -0.72 | 0.007 | 0.016 | 13 | Yes |
| GPR84 | Leptin | metabolic | -0.747 | 0.00333 | 0.016 | 13 | Yes |
| Ffar4 | MCP-1 | cytokine | -0.736 | 0.006 | 0.017 | 13 | Yes |
| Ffar1 | IL-6R | receptor | -0.707 | 0.01 | 0.018 | 13 | Yes |
| Ffar1 | TNF-a | cytokine | -0.692 | 0.009 | 0.018 | 13 | Yes |
| Ffar1 | IL-10 | cytokine | -0.676 | 0.01 | 0.018 | 13 | Yes |
| FP number | Waist/length ratio | metabolic | -0.737 | 0.00409 | 0.019 | 13 | Yes |
| Ffar4 | Leptin | metabolic | -0.714 | 0.008 | 0.02 | 13 | Yes |
| Ffar4 | TNF-a | cytokine | -0.703 | 0.01 | 0.02 | 13 | Yes |
| Ffar4 | Relative abdominal fat mass | metabolic | -0.698 | 0.009 | 0.02 | 13 | Yes |
| GPR84 | TNF-a | cytokine | -0.703 | 0.009 | 0.02 | 13 | Yes |
| Ffar1 | IL-23 | cytokine | -0.659 | 0.014 | 0.021 | 13 | Yes |
| Ffar1 | IL-1b | cytokine | -0.654 | 0.014 | 0.021 | 13 | Yes |
| Ffar1 | IFN-b | cytokine | -0.654 | 0.015 | 0.021 | 13 | Yes |
| FP area | Body Weight | metabolic | -0.725 | 0.00502 | 0.021 | 13 | Yes |
| CD36 | IL-1b | cytokine | -0.698 | 0.01 | 0.022 | 13 | Yes |
| GPR84 | IL-1b | cytokine | -0.681 | 0.013 | 0.024 | 13 | Yes |
| GPR84 | QUICKI | metabolic | 0.67 | 0.014 | 0.024 | 13 | Yes |
| Ffar4 | Waist/length ratio | metabolic | -0.681 | 0.014 | 0.026 | 13 | Yes |
| FP area | IL-6R | receptor | -0.704 | 0.0072 | 0.027 | 13 | Yes |
| Tas1r2 | IL-1b | cytokine | -0.703 | 0.00732 | 0.027 | 13 | Yes |
| CD36 | QUICKI | metabolic | 0.676 | 0.016 | 0.027 | 13 | Yes |
| CD36 | IL-6R | receptor | -0.633 | 0.016 | 0.027 | 13 | Yes |
| Ffar4 | QUICKI | metabolic | 0.659 | 0.015 | 0.027 | 13 | Yes |
| FP area | TNF-R2 | receptor | 0.698 | 0.008 | 0.028 | 13 | Yes |
| GPR84 | IL-23 | cytokine | -0.676 | 0.018 | 0.028 | 13 | Yes |
| GPR84 | IL-10 | cytokine | -0.665 | 0.018 | 0.028 | 13 | Yes |
| Tas1r3 | TNF-R2 | receptor | -0.692 | 0.00873 | 0.029 | 13 | Yes |
| GPR84 | IL-6R | receptor | -0.633 | 0.022 | 0.032 | 13 | Yes |
| Ffar4 | IL-1b | cytokine | -0.692 | 0.02 | 0.034 | 13 | Yes |
| Tas1r2 | IL-6R | receptor | -0.608 | 0.02 | 0.034 | 13 | Yes |
| CD36 | IL-10 | cytokine | -0.621 | 0.025 | 0.037 | 13 | Yes |
| CD36 | IL-23 | cytokine | -0.61 | 0.025 | 0.037 | 13 | Yes |
| Ffar1 | IL-27 | cytokine | -0.593 | 0.028 | 0.037 | 13 | Yes |
| Tas1r2 | MCP-1 | cytokine | -0.665 | 0.0132 | 0.037 | 13 | Yes |
| Ffar4 | IFN-b | cytokine | -0.659 | 0.0142 | 0.039 | 13 | Yes |
| FP number | Body Weight | metabolic | -0.612 | 0.0261 | 0.039 | 13 | Yes |
| Ffar1 | IFN-g | cytokine | -0.588 | 0.031 | 0.04 | 13 | Yes |
| Tas1r2 | Body Weight | metabolic | -0.676 | 0.022 | 0.042 | 13 | Yes |
| Tas1r2 | IL-17A | cytokine | -0.648 | 0.0165 | 0.043 | 13 | Yes |
| GPR84 | IFN-b | cytokine | -0.621 | 0.032 | 0.045 | 13 | Yes |
| GPR84 | IFN-g | cytokine | -0.61 | 0.034 | 0.045 | 13 | Yes |
| Tas1r2 | Waist/length ratio | metabolic | -0.582 | 0.038 | 0.045 | 13 | Yes |
| FP number | Leptin | metabolic | -0.643 | 0.0178 | 0.045 | 13 | Yes |
| Tas1r3 | Waist/length ratio | metabolic | 0.643 | 0.0178 | 0.045 | 13 | Yes |
| Ffar4 | IFN-g | cytokine | -0.593 | 0.037 | 0.048 | 13 | Yes |
| Tas1r2 | IL-1a | cytokine | -0.637 | 0.0191 | 0.048 | 13 | Yes |
| CD36 | IFN-g | cytokine | -0.582 | 0.037 | 0.049 | 13 | Yes |
| CD36 | IFN-b | cytokine | -0.566 | 0.041 | 0.049 | 13 | Yes |
| Ffar4 | IL-23 | cytokine | -0.621 | 0.032 | 0.049 | 13 | Yes |
| FP number | Relative abdominal fat mass | metabolic | -0.599 | 0.0307 | 0.049 | 13 | Yes |
| Tas1r2 | TNF-R2 | receptor | 0.632 | 0.0205 | 0.05 | 13 | Yes |
| Tas1r2 | TNF-a | cytokine | -0.626 | 0.022 | 0.05 | 13 | Yes |
| GPR84 | IL-27 | cytokine | -0.593 | 0.047 | 0.058 | 13 | No |
| Ffar4 | IL-27 | cytokine | -0.577 | 0.044 | 0.062 | 13 | No |
| Ffar1 | IL-6 | cytokine | -0.478 | 0.069 | 0.081 | 13 | No |
| CD36 | IL-27 | cytokine | -0.505 | 0.068 | 0.083 | 13 | No |
| Ffar4 | IL-6 | cytokine | -0.516 | 0.067 | 0.085 | 13 | No |
| FP number | IL-6 | cytokine | -0.563 | 0.0453 | 0.091 | 13 | No |
| Ffar1 | GM-CSF | cytokine | -0.489 | 0.087 | 0.094 | 13 | No |
| FP number | TNF-R2 | receptor | 0.552 | 0.0506 | 0.099 | 13 | No |
| GPR84 | GM-CSF | cytokine | -0.511 | 0.091 | 0.102 | 13 | No |
| GPR84 | IL-6 | cytokine | -0.505 | 0.099 | 0.107 | 13 | No |
| Tas1r2 | IL-6 | cytokine | -0.555 | 0.056 | 0.112 | 13 | No |
| Tas1r2 | IL-27 | cytokine | -0.555 | 0.06 | 0.113 | 13 | No |
| FP number | IL-6R | receptor | -0.526 | 0.0647 | 0.12 | 13 | No |
| FP area | Leptin | metabolic | -0.522 | 0.0673 | 0.123 | 13 | No |
| CD36 | IL-6 | cytokine | -0.451 | 0.118 | 0.127 | 13 | No |
| CD36 | GM-CSF | cytokine | -0.44 | 0.116 | 0.127 | 13 | No |
| Ffar4 | IL-6R | receptor | -0.484 | 0.111 | 0.135 | 13 | No |
| FP number | non-HDL | metabolic | -0.504 | 0.079 | 0.137 | 13 | No |
| Ffar4 | GM-CSF | cytokine | -0.478 | 0.127 | 0.148 | 13 | No |
| Tas1r2 | IL-23 | cytokine | -0.522 | 0.088 | 0.154 | 13 | No |
| Tas1r2 | Leptin | metabolic | -0.473 | 0.109 | 0.161 | 13 | No |
| Tas1r2 | QUICKI | metabolic | 0.462 | 0.107 | 0.161 | 13 | No |
| FP area | QUICKI | metabolic | 0.473 | 0.103 | 0.166 | 13 | No |
| FP area | Relative abdominal fat mass | metabolic | -0.445 | 0.128 | 0.194 | 13 | No |
| Tas1r3 | Body Weight | metabolic | 0.555 | 0.053 | 0.203 | 13 | No |
| Tas1r3 | IL-6R | receptor | 0.547 | 0.051 | 0.203 | 13 | No |
| FP area | IL-17A | cytokine | -0.434 | 0.138 | 0.208 | 13 | No |
| FP area | IL-1a | cytokine | -0.385 | 0.194 | 0.27 | 13 | No |
| Tas1r2 | Relative abdominal fat mass | metabolic | -0.379 | 0.202 | 0.282 | 13 | No |
| FP area | IL-6 | cytokine | -0.374 | 0.209 | 0.283 | 13 | No |
| Tas1r3 | Relative abdominal fat mass | metabolic | 0.467 | 0.11 | 0.293 | 13 | No |
| Tas1r3 | Leptin | metabolic | 0.462 | 0.115 | 0.293 | 13 | No |
| FP number | IL-17A | cytokine | -0.359 | 0.229 | 0.306 | 13 | No |
| FP area | TNF-a | cytokine | -0.352 | 0.239 | 0.315 | 13 | No |
| Tas1r2 | IFN-b | cytokine | -0.396 | 0.242 | 0.323 | 13 | No |
| Tas1r2 | IL-10 | cytokine | -0.341 | 0.258 | 0.329 | 13 | No |
| CD36 | TNF-R1 | receptor | 0.297 | 0.32 | 0.332 | 13 | No |
| FP area | MCP-1 | cytokine | -0.335 | 0.263 | 0.342 | 13 | No |
| FP number | IL-1a | cytokine | -0.331 | 0.269 | 0.349 | 13 | No |
| Tas1r3 | IL-17A | cytokine | 0.412 | 0.163 | 0.352 | 13 | No |
| Tas1r3 | non-HDL | metabolic | 0.399 | 0.177 | 0.354 | 13 | No |
| GPR84 | TNF-R1 | receptor | 0.28 | 0.343 | 0.356 | 13 | No |
| Ffar4 | TNF-R1 | receptor | 0.275 | 0.367 | 0.367 | 13 | No |
| Ffar4 | non-HDL | metabolic | -0.231 | 0.359 | 0.367 | 13 | No |
| FP area | IL-1b | cytokine | -0.308 | 0.306 | 0.386 | 13 | No |
| FP number | IL-10 | cytokine | -0.306 | 0.309 | 0.387 | 13 | No |
| Tas1r2 | IFN-g | cytokine | -0.319 | 0.343 | 0.401 | 13 | No |
| FP number | MCP-1 | cytokine | -0.281 | 0.352 | 0.431 | 13 | No |
| FP number | TNF-a | cytokine | -0.279 | 0.357 | 0.434 | 13 | No |
| GPR84 | non-HDL | metabolic | -0.223 | 0.439 | 0.439 | 13 | No |
| FP area | IFN-b | cytokine | -0.269 | 0.374 | 0.448 | 13 | No |
| FP area | non-HDL | metabolic | -0.267 | 0.378 | 0.452 | 13 | No |
| Tas1r2 | GM-CSF | cytokine | -0.291 | 0.404 | 0.453 | 13 | No |
| FP area | IL-10 | cytokine | -0.264 | 0.384 | 0.456 | 13 | No |
| Tas1r3 | TNF-a | cytokine | 0.335 | 0.263 | 0.461 | 13 | No |
| Tas1r3 | IL-10 | cytokine | 0.33 | 0.271 | 0.461 | 13 | No |
| Tas1r3 | IL-1a | cytokine | 0.324 | 0.28 | 0.461 | 13 | No |
| Ffar1 | TNF-R1 | receptor | 0.209 | 0.47 | 0.487 | 13 | No |
| FP area | IL-23 | cytokine | -0.231 | 0.448 | 0.511 | 13 | No |
| FP area | IL-27 | cytokine | -0.231 | 0.448 | 0.511 | 13 | No |
| FP number | IFN-g | cytokine | -0.234 | 0.441 | 0.511 | 13 | No |
| Ffar1 | non-HDL | metabolic | -0.154 | 0.517 | 0.517 | 13 | No |
| Tas1r3 | MCP-1 | cytokine | 0.275 | 0.363 | 0.55 | 13 | No |
| Tas1r3 | IL-1b | cytokine | 0.269 | 0.373 | 0.55 | 13 | No |
| FP number | IL-1b | cytokine | -0.21 | 0.492 | 0.552 | 13 | No |
| FP area | TNF-R1 | receptor | 0.198 | 0.517 | 0.575 | 13 | No |
| FP number | TNF-R1 | receptor | 0.193 | 0.527 | 0.584 | 13 | No |
| CD36 | non-HDL | metabolic | -0.193 | 0.592 | 0.592 | 13 | No |
| Tas1r2 | non-HDL | metabolic | -0.121 | 0.6 | 0.613 | 13 | No |
| Tas1r3 | QUICKI | metabolic | -0.253 | 0.458 | 0.642 | 13 | No |
| Tas1r3 | IL-23 | cytokine | 0.192 | 0.529 | 0.674 | 13 | No |
| FP area | IFN-g | cytokine | -0.143 | 0.642 | 0.687 | 13 | No |
| FP number | IL-23 | cytokine | -0.143 | 0.64 | 0.687 | 13 | No |
| Tas1r3 | IL-27 | cytokine | 0.159 | 0.604 | 0.735 | 13 | No |
| FP area | GM-CSF | cytokine | -0.115 | 0.707 | 0.744 | 13 | No |
| FP number | IL-27 | cytokine | -0.102 | 0.74 | 0.776 | 13 | No |
| FP number | GM-CSF | cytokine | 0.091 | 0.767 | 0.797 | 13 | No |
| Tas1r3 | TNF-R1 | receptor | 0.082 | 0.789 | 0.813 | 13 | No |
| Tas1r3 | IFN-g | cytokine | 0.099 | 0.751 | 0.856 | 13 | No |
| Tas1r3 | IFN-b | cytokine | 0.093 | 0.765 | 0.856 | 13 | No |
| FP number | IFN-b | cytokine | -0.063 | 0.837 | 0.86 | 13 | No |
| Tas1r2 | TNF-R1 | receptor | 0.198 | 0.803 | 0.864 | 13 | No |
| Tas1r3 | GM-CSF | cytokine | -0.033 | 0.92 | 0.949 | 13 | No |
| Tas1r3 | IL-6 | cytokine | 0.022 | 0.949 | 0.949 | 13 | No |

***Table S4:*** *Edge Table Jejunum.*

| **Anchor (taste/FP)** | **Partner** | **Partner Category** | **Spearman r** | **Raw p-value** | **FDR-adjusted p-value** | **n** | **In network (FDR<0.05, \|r\|>0.5)** |
| --- | --- | --- | --- | --- | --- | --- | --- |
| GPR84 | TNF-R2 | receptor | 0.934 | <0.0001 | 0.0001784 | 13 | Yes |
| CD36 | TNF-R2 | receptor | 0.912 | <0.0001 | 0.0003772 | 13 | Yes |
| Ffar1 | TNF-R1 | receptor | -0.857 | 0.0003611 | 0.002 | 13 | Yes |
| Ffar1 | TNF-R2 | receptor | 0.846 | 0.001 | 0.003 | 13 | Yes |
| CD36 | TNF-R1 | receptor | -0.83 | 0.001 | 0.004 | 13 | Yes |
| FP area | TNF-R2 | receptor | 0.852 | 0.000428 | 0.004 | 13 | Yes |
| CD36 | Body Weight | metabolic | -0.769 | 0.003 | 0.012 | 13 | Yes |
| CD36 | Waist/length ratio | metabolic | -0.769 | 0.003 | 0.012 | 13 | Yes |
| FP area | Waist/length ratio | metabolic | -0.786 | 0.002 | 0.012 | 13 | Yes |
| GPR84 | Waist/length ratio | metabolic | -0.78 | 0.003 | 0.015 | 13 | Yes |
| FP area | TNF-R1 | receptor | -0.753 | 0.004 | 0.016 | 13 | Yes |
| FP number | QUICKI | metabolic | 0.761 | 0.0025 | 0.016 | 13 | Yes |
| GPR84 | Body Weight | metabolic | -0.736 | 0.005 | 0.021 | 13 | Yes |
| FP area | Body Weight | metabolic | -0.725 | 0.007 | 0.022 | 13 | Yes |
| FP number | Waist/length ratio | metabolic | -0.737 | 0.00409 | 0.022 | 13 | Yes |
| GPR84 | TNF-R1 | receptor | -0.703 | 0.008 | 0.027 | 13 | Yes |
| CD36 | Leptin | metabolic | -0.709 | 0.009 | 0.029 | 13 | Yes |
| Ffar1 | Waist/length ratio | metabolic | -0.703 | 0.009 | 0.036 | 13 | Yes |
| Ffar4 | TNF-R2 | receptor | 0.654 | 0.0153 | 0.038 | 13 | Yes |
| FP number | Body Weight | metabolic | -0.612 | 0.0261 | 0.039 | 13 | Yes |
| FP number | TNF-R2 | receptor | 0.654 | 0.018 | 0.042 | 13 | Yes |
| FP number | Leptin | metabolic | -0.643 | 0.0178 | 0.045 | 13 | Yes |
| Ffar1 | Leptin | metabolic | -0.654 | 0.018 | 0.046 | 13 | Yes |
| FP number | Relative abdominal fat mass | metabolic | -0.599 | 0.0307 | 0.048 | 13 | Yes |
| GPR84 | Leptin | metabolic | -0.632 | 0.025 | 0.048 | 13 | Yes |
| Ffar1 | Body Weight | metabolic | -0.67 | 0.015 | 0.05 | 13 | Yes |
| GPR84 | IL-17A | cytokine | -0.593 | 0.033 | 0.089 | 13 | No |
| CD36 | IL-17A | cytokine | -0.599 | 0.034 | 0.092 | 13 | No |
| GPR84 | Relative abdominal fat mass | metabolic | -0.549 | 0.06 | 0.134 | 13 | No |
| GPR84 | IL-1a | cytokine | -0.533 | 0.057 | 0.134 | 13 | No |
| FP number | IL-6 | cytokine | -0.563 | 0.0453 | 0.136 | 13 | No |
| GPR84 | TNF-a | cytokine | -0.5 | 0.071 | 0.137 | 13 | No |
| CD36 | IL-1a | cytokine | -0.544 | 0.058 | 0.14 | 13 | No |
| CD36 | Relative abdominal fat mass | metabolic | -0.527 | 0.067 | 0.14 | 13 | No |
| CD36 | TNF-a | cytokine | -0.527 | 0.067 | 0.14 | 13 | No |
| GPR84 | MCP-1 | cytokine | -0.473 | 0.091 | 0.164 | 13 | No |
| FP number | TNF-R1 | receptor | -0.535 | 0.0595 | 0.165 | 13 | No |
| Tas1r3 | IL-6 | cytokine | 0.527 | 0.064 | 0.169 | 13 | No |
| CD36 | MCP-1 | cytokine | -0.495 | 0.089 | 0.171 | 13 | No |
| Ffar1 | IL-17A | cytokine | -0.516 | 0.074 | 0.182 | 13 | No |
| GPR84 | QUICKI | metabolic | 0.489 | 0.11 | 0.186 | 13 | No |
| CD36 | IL-1b | cytokine | -0.473 | 0.106 | 0.19 | 13 | No |
| FP area | Leptin | metabolic | -0.522 | 0.071 | 0.191 | 13 | No |
| CD36 | QUICKI | metabolic | 0.467 | 0.123 | 0.196 | 13 | No |
| CD36 | IL-10 | cytokine | -0.456 | 0.12 | 0.196 | 13 | No |
| FP number | non-HDL | metabolic | -0.504 | 0.079 | 0.198 | 13 | No |
| Ffar1 | IL-1a | cytokine | -0.484 | 0.097 | 0.202 | 13 | No |
| Ffar1 | Relative abdominal fat mass | metabolic | -0.467 | 0.11 | 0.202 | 13 | No |
| Ffar1 | QUICKI | metabolic | 0.462 | 0.113 | 0.202 | 13 | No |
| Ffar1 | TNF-a | cytokine | -0.456 | 0.12 | 0.202 | 13 | No |
| Ffar1 | IL-10 | cytokine | -0.456 | 0.12 | 0.202 | 13 | No |
| Tas1r3 | TNF-R1 | receptor | -0.489 | 0.0899 | 0.214 | 13 | No |
| GPR84 | IL-10 | cytokine | -0.418 | 0.153 | 0.221 | 13 | No |
| GPR84 | IL-23 | cytokine | -0.407 | 0.155 | 0.221 | 13 | No |
| GPR84 | IL-1b | cytokine | -0.401 | 0.147 | 0.221 | 13 | No |
| Ffar1 | MCP-1 | cytokine | -0.423 | 0.152 | 0.241 | 13 | No |
| FP area | QUICKI | metabolic | 0.473 | 0.1 | 0.245 | 13 | No |
| CD36 | IL-23 | cytokine | -0.401 | 0.176 | 0.25 | 13 | No |
| GPR84 | IL-27 | cytokine | -0.379 | 0.193 | 0.261 | 13 | No |
| Ffar1 | non-HDL | metabolic | -0.396 | 0.18 | 0.27 | 13 | No |
| CD36 | IL-27 | cytokine | -0.374 | 0.209 | 0.283 | 13 | No |
| FP area | Relative abdominal fat mass | metabolic | -0.445 | 0.13 | 0.292 | 13 | No |
| FP area | IL-17A | cytokine | -0.434 | 0.14 | 0.292 | 13 | No |
| Ffar1 | IL-1b | cytokine | -0.374 | 0.209 | 0.298 | 13 | No |
| Ffar1 | IFN-b | cytokine | -0.363 | 0.224 | 0.302 | 13 | No |
| CD36 | non-HDL | metabolic | -0.352 | 0.236 | 0.304 | 13 | No |
| Ffar1 | IL-23 | cytokine | -0.346 | 0.247 | 0.313 | 13 | No |
| Ffar1 | IL-27 | cytokine | -0.341 | 0.255 | 0.313 | 13 | No |
| CD36 | IFN-b | cytokine | -0.33 | 0.271 | 0.327 | 13 | No |
| CD36 | IL-6R | receptor | -0.323 | 0.279 | 0.327 | 13 | No |
| Tas1r3 | IL-27 | cytokine | 0.401 | 0.174 | 0.33 | 13 | No |
| GPR84 | IL-6 | cytokine | -0.346 | 0.26 | 0.335 | 13 | No |
| Tas1r3 | IL-1a | cytokine | 0.39 | 0.188 | 0.344 | 13 | No |
| GPR84 | non-HDL | metabolic | -0.305 | 0.302 | 0.371 | 13 | No |
| FP area | IL-1a | cytokine | -0.385 | 0.196 | 0.377 | 13 | No |
| FP area | IL-6 | cytokine | -0.374 | 0.209 | 0.377 | 13 | No |
| FP number | IL-17A | cytokine | -0.359 | 0.229 | 0.383 | 13 | No |
| GPR84 | IFN-g | cytokine | -0.286 | 0.336 | 0.389 | 13 | No |
| GPR84 | IFN-b | cytokine | -0.264 | 0.346 | 0.389 | 13 | No |
| Tas1r3 | Waist/length ratio | metabolic | -0.341 | 0.255 | 0.403 | 13 | No |
| FP area | TNF-a | cytokine | -0.352 | 0.239 | 0.404 | 13 | No |
| Ffar1 | IL-6 | cytokine | -0.28 | 0.353 | 0.415 | 13 | No |
| FP area | MCP-1 | cytokine | -0.335 | 0.263 | 0.418 | 13 | No |
| FP number | IL-1a | cytokine | -0.331 | 0.269 | 0.422 | 13 | No |
| Tas1r3 | TNF-a | cytokine | 0.324 | 0.28 | 0.429 | 13 | No |
| Tas1r3 | MCP-1 | cytokine | 0.308 | 0.306 | 0.458 | 13 | No |
| FP area | IL-1b | cytokine | -0.308 | 0.306 | 0.459 | 13 | No |
| FP number | IL-10 | cytokine | -0.306 | 0.309 | 0.46 | 13 | No |
| FP area | IFN-b | cytokine | -0.269 | 0.373 | 0.471 | 13 | No |
| FP area | non-HDL | metabolic | -0.267 | 0.375 | 0.471 | 13 | No |
| FP area | IL-10 | cytokine | -0.264 | 0.383 | 0.471 | 13 | No |
| CD36 | IL-6 | cytokine | -0.231 | 0.448 | 0.484 | 13 | No |
| Tas1r3 | IFN-g | cytokine | 0.291 | 0.334 | 0.484 | 13 | No |
| FP number | MCP-1 | cytokine | -0.281 | 0.352 | 0.499 | 13 | No |
| CD36 | IFN-g | cytokine | -0.214 | 0.482 | 0.5 | 13 | No |
| FP number | TNF-a | cytokine | -0.279 | 0.357 | 0.501 | 13 | No |
| FP area | IL-23 | cytokine | -0.231 | 0.448 | 0.504 | 13 | No |
| FP area | IL-27 | cytokine | -0.231 | 0.448 | 0.504 | 13 | No |
| GPR84 | IL-6R | receptor | -0.212 | 0.476 | 0.514 | 13 | No |
| Tas1r3 | Leptin | metabolic | -0.253 | 0.405 | 0.539 | 13 | No |
| FP number | IFN-g | cytokine | -0.234 | 0.441 | 0.565 | 13 | No |
| Tas1r3 | IL-6R | receptor | 0.234 | 0.442 | 0.565 | 13 | No |
| Ffar1 | IFN-g | cytokine | -0.192 | 0.529 | 0.572 | 13 | No |
| Tas1r3 | IL-23 | cytokine | 0.225 | 0.459 | 0.573 | 13 | No |
| FP number | IL-1b | cytokine | -0.21 | 0.492 | 0.604 | 13 | No |
| Tas1r3 | GM-CSF | cytokine | 0.203 | 0.505 | 0.618 | 13 | No |
| Tas1r3 | non-HDL | metabolic | -0.198 | 0.517 | 0.627 | 13 | No |
| Tas1r3 | Relative abdominal fat mass | metabolic | -0.192 | 0.529 | 0.635 | 13 | No |
| Tas1r3 | IL-1b | cytokine | 0.192 | 0.529 | 0.635 | 13 | No |
| GPR84 | GM-CSF | cytokine | -0.132 | 0.639 | 0.639 | 13 | No |
| FP number | IL-6R | receptor | 0.166 | 0.587 | 0.691 | 13 | No |
| FP area | IFN-g | cytokine | -0.143 | 0.643 | 0.694 | 13 | No |
| FP area | GM-CSF | cytokine | -0.115 | 0.71 | 0.737 | 13 | No |
| FP number | IL-23 | cytokine | -0.143 | 0.64 | 0.737 | 13 | No |
| Tas1r3 | TNF-R2 | receptor | 0.137 | 0.655 | 0.741 | 13 | No |
| FP area | IL-6R | receptor | 0.098 | 0.75 | 0.75 | 13 | No |
| Tas1r3 | Body Weight | metabolic | -0.126 | 0.681 | 0.759 | 13 | No |
| Tas1r3 | IFN-b | cytokine | 0.115 | 0.707 | 0.782 | 13 | No |
| FP number | IL-27 | cytokine | -0.102 | 0.74 | 0.808 | 13 | No |
| FP number | GM-CSF | cytokine | 0.091 | 0.767 | 0.824 | 13 | No |
| Tas1r3 | IL-10 | cytokine | 0.088 | 0.775 | 0.828 | 13 | No |
| Ffar4 | TNF-R1 | receptor | -0.363 | 0.224 | 0.834 | 13 | No |
| Ffar4 | Waist/length ratio | metabolic | -0.346 | 0.247 | 0.834 | 13 | No |
| Ffar1 | IL-6R | receptor | -0.075 | 0.807 | 0.838 | 13 | No |
| CD36 | GM-CSF | cytokine | -0.055 | 0.863 | 0.863 | 13 | No |
| FP number | IFN-b | cytokine | -0.063 | 0.837 | 0.879 | 13 | No |
| Tas1r3 | QUICKI | metabolic | -0.049 | 0.873 | 0.904 | 13 | No |
| Ffar1 | GM-CSF | cytokine | -0.038 | 0.906 | 0.906 | 13 | No |
| Ffar4 | IL-6 | cytokine | -0.302 | 0.315 | 0.941 | 13 | No |
| Ffar4 | IL-6R | receptor | 0.273 | 0.363 | 0.941 | 13 | No |
| Ffar4 | IFN-g | cytokine | 0.264 | 0.383 | 0.941 | 13 | No |
| Ffar4 | GM-CSF | cytokine | 0.187 | 0.541 | 0.949 | 13 | No |
| Ffar4 | Body Weight | metabolic | -0.165 | 0.591 | 0.949 | 13 | No |
| Ffar4 | IFN-b | cytokine | 0.159 | 0.604 | 0.949 | 13 | No |
| Ffar4 | IL-1b | cytokine | 0.148 | 0.63 | 0.949 | 13 | No |
| Ffar4 | non-HDL | metabolic | -0.14 | 0.646 | 0.949 | 13 | No |
| Ffar4 | IL-27 | cytokine | -0.11 | 0.723 | 0.949 | 13 | No |
| Ffar4 | IL-10 | cytokine | 0.104 | 0.737 | 0.949 | 13 | No |
| Ffar4 | Relative abdominal fat mass | metabolic | 0.099 | 0.751 | 0.949 | 13 | No |
| Ffar4 | IL-23 | cytokine | 0.099 | 0.751 | 0.949 | 13 | No |
| Ffar4 | MCP-1 | cytokine | 0.082 | 0.793 | 0.949 | 13 | No |
| Ffar4 | TNF-a | cytokine | 0.049 | 0.878 | 0.949 | 13 | No |
| Ffar4 | Leptin | metabolic | 0.033 | 0.92 | 0.949 | 13 | No |
| Ffar4 | QUICKI | metabolic | 0.027 | 0.895 | 0.949 | 13 | No |
| Ffar4 | IL-1a | cytokine | -0.022 | 0.949 | 0.949 | 13 | No |
| Ffar4 | IL-17A | cytokine | 0.022 | 0.949 | 0.949 | 13 | No |
| Tas1r3 | IL-17A | cytokine | 0.016 | 0.957 | 0.965 | 13 | No |

***Table S5:*** *Edge Table Stomach.*

| **Anchor (taste/FP)** | **Partner** | **Partner Category** | **Spearman r** | **Raw p-value** | **FDR-adjusted p-value** | **n** | **In network (FDR<0.05, \|r\|>0.5)** |
| --- | --- | --- | --- | --- | --- | --- | --- |
| FP area | Waist/length ratio | metabolic | -0.786 | 0.00145 | 0.017 | 13 | Yes |
| FP number | QUICKI | metabolic | 0.761 | 0.0025 | 0.023 | 13 | Yes |
| Tas1r2 | IL-6 | cytokine | 0.687 | 0.00951 | 0.030 | 13 | Yes |
| FP number | Waist/length ratio | metabolic | -0.737 | 0.00409 | 0.033 | 13 | Yes |
| FP area | Body Weight | metabolic | -0.725 | 0.00502 | 0.038 | 13 | Yes |
| Tas1r2 | QUICKI | metabolic | -0.626 | 0.025 | 0.038 | 13 | No |
| FP number | Body Weight | metabolic | -0.612 | 0.0261 | 0.039 | 13 | Yes |
| FP number | Leptin | metabolic | -0.643 | 0.0178 | 0.045 | 13 | Yes |
| FP number | Relative abdominal fat mass | metabolic | -0.599 | 0.0307 | 0.048 | 13 | Yes |
| Tas1r2 | Relative abdominal fat mass | metabolic | 0.593 | 0.036 | 0.05 | 13 | No |
| FP number | IL-6 | cytokine | -0.563 | 0.0453 | 0.182 | 13 | No |
| Tas1r2 | IL-10 | cytokine | 0.593 | 0.036 | 0.23 | 13 | No |
| FP area | Leptin | metabolic | -0.522 | 0.0673 | 0.246 | 13 | No |
| FP number | non-HDL | metabolic | -0.504 | 0.079 | 0.276 | 13 | No |
| FP area | QUICKI | metabolic | 0.473 | 0.103 | 0.324 | 13 | No |
| Tas1r2 | Waist/length ratio | metabolic | 0.511 | 0.078 | 0.34 | 13 | No |
| Tas1r2 | IL-1a | cytokine | 0.489 | 0.093 | 0.34 | 13 | No |
| FP area | Relative abdominal fat mass | metabolic | -0.445 | 0.128 | 0.362 | 13 | No |
| Tas1r2 | Leptin | metabolic | 0.456 | 0.12 | 0.373 | 13 | No |
| FP area | IL-17A | cytokine | -0.434 | 0.138 | 0.382 | 13 | No |
| Tas1r2 | IL-17A | cytokine | 0.407 | 0.17 | 0.405 | 13 | No |
| Tas1r2 | IFN-g | cytokine | 0.39 | 0.189 | 0.405 | 13 | No |
| Tas1r2 | IL-27 | cytokine | 0.363 | 0.224 | 0.405 | 13 | No |
| Tas1r2 | IFN-b | cytokine | 0.363 | 0.224 | 0.405 | 13 | No |
| Tas1r2 | TNF-a | cytokine | 0.357 | 0.232 | 0.405 | 13 | No |
| Tas1r2 | MCP-1 | cytokine | 0.357 | 0.232 | 0.405 | 13 | No |
| FP area | IL-1a | cytokine | -0.385 | 0.194 | 0.451 | 13 | No |
| Tas1r3 | Relative abdominal fat mass | metabolic | 0.632 | 0.024 | 0.46 | 13 | No |
| Tas1r3 | TNF-R2 | receptor | -0.599 | 0.033 | 0.46 | 13 | No |
| FP area | IL-6 | cytokine | -0.374 | 0.209 | 0.465 | 13 | No |
| Tas1r2 | Body Weight | metabolic | 0.319 | 0.289 | 0.475 | 13 | No |
| FP number | IL-17A | cytokine | -0.359 | 0.229 | 0.486 | 13 | No |
| FP area | TNF-a | cytokine | -0.352 | 0.239 | 0.492 | 13 | No |
| FP area | MCP-1 | cytokine | -0.335 | 0.263 | 0.516 | 13 | No |
| Tas1r2 | non-HDL | metabolic | 0.289 | 0.336 | 0.523 | 13 | No |
| FP number | IL-1a | cytokine | -0.331 | 0.269 | 0.526 | 13 | No |
| FP area | IL-6R | receptor | -0.319 | 0.289 | 0.535 | 13 | No |
| Tas1r3 | IFN-g | cytokine | 0.544 | 0.058 | 0.543 | 13 | No |
| FP area | IL-1b | cytokine | -0.308 | 0.306 | 0.551 | 13 | No |
| FP number | IL-10 | cytokine | -0.306 | 0.309 | 0.552 | 13 | No |
| FP number | MCP-1 | cytokine | -0.281 | 0.352 | 0.584 | 13 | No |
| FP number | TNF-a | cytokine | -0.279 | 0.357 | 0.586 | 13 | No |
| Tas1r2 | IL-1b | cytokine | 0.214 | 0.482 | 0.601 | 13 | No |
| Tas1r2 | IL-23 | cytokine | 0.209 | 0.493 | 0.601 | 13 | No |
| Tas1r3 | TNF-R1 | receptor | -0.423 | 0.141 | 0.601 | 13 | No |
| Tas1r3 | IL-10 | cytokine | 0.39 | 0.189 | 0.601 | 13 | No |
| Tas1r3 | GM-CSF | cytokine | 0.374 | 0.209 | 0.601 | 13 | No |
| Tas1r3 | IL-17A | cytokine | 0.341 | 0.255 | 0.601 | 13 | No |
| Tas1r3 | QUICKI | metabolic | -0.324 | 0.278 | 0.601 | 13 | No |
| Tas1r3 | TNF-a | cytokine | 0.308 | 0.306 | 0.601 | 13 | No |
| Tas1r3 | Waist/length ratio | metabolic | 0.286 | 0.344 | 0.601 | 13 | No |
| Tas1r3 | MCP-1 | cytokine | 0.286 | 0.344 | 0.601 | 13 | No |
| Tas1r3 | non-HDL | metabolic | 0.267 | 0.375 | 0.601 | 13 | No |
| Tas1r3 | IFN-b | cytokine | 0.242 | 0.426 | 0.601 | 13 | No |
| Tas1r3 | IL-1a | cytokine | 0.236 | 0.437 | 0.601 | 13 | No |
| Tas1r3 | IL-1b | cytokine | 0.225 | 0.459 | 0.601 | 13 | No |
| Tas1r3 | Leptin | metabolic | 0.209 | 0.493 | 0.601 | 13 | No |
| Tas1r3 | IL-23 | cytokine | 0.209 | 0.493 | 0.601 | 13 | No |
| Tas1r3 | IL-6R | receptor | -0.192 | 0.49 | 0.601 | 13 | No |
| FP area | IFN-b | cytokine | -0.269 | 0.374 | 0.607 | 13 | No |
| FP area | non-HDL | metabolic | -0.267 | 0.378 | 0.609 | 13 | No |
| FP area | IL-10 | cytokine | -0.264 | 0.384 | 0.614 | 13 | No |
| Tas1r2 | TNF-R1 | receptor | -0.181 | 0.538 | 0.627 | 13 | No |
| Tas1r3 | Body Weight | metabolic | 0.176 | 0.566 | 0.634 | 13 | No |
| Tas1r3 | IL-6 | cytokine | 0.176 | 0.566 | 0.634 | 13 | No |
| Tas1r2 | TNF-R2 | receptor | -0.187 | 0.606 | 0.653 | 13 | No |
| Tas1r2 | GM-CSF | cytokine | 0.165 | 0.591 | 0.653 | 13 | No |
| FP area | IL-23 | cytokine | -0.231 | 0.448 | 0.654 | 13 | No |
| FP area | IL-27 | cytokine | -0.231 | 0.448 | 0.654 | 13 | No |
| FP number | IFN-g | cytokine | -0.234 | 0.441 | 0.654 | 13 | No |
| FP number | IL-1b | cytokine | -0.21 | 0.492 | 0.689 | 13 | No |
| CD36 | Body Weight | metabolic | -0.385 | 0.196 | 0.697 | 13 | No |
| CD36 | IFN-g | cytokine | -0.363 | 0.224 | 0.697 | 13 | No |
| CD36 | Relative abdominal fat mass | metabolic | -0.352 | 0.239 | 0.697 | 13 | No |
| CD36 | TNF-R2 | receptor | 0.352 | 0.26 | 0.697 | 13 | No |
| CD36 | IL-1b | cytokine | -0.341 | 0.255 | 0.697 | 13 | No |
| CD36 | TNF-R1 | receptor | -0.335 | 0.252 | 0.697 | 13 | No |
| CD36 | QUICKI | metabolic | 0.33 | 0.296 | 0.697 | 13 | No |
| CD36 | MCP-1 | cytokine | -0.308 | 0.306 | 0.697 | 13 | No |
| CD36 | IL-17A | cytokine | -0.308 | 0.306 | 0.697 | 13 | No |
| CD36 | Leptin | metabolic | -0.302 | 0.315 | 0.697 | 13 | No |
| CD36 | TNF-a | cytokine | -0.28 | 0.353 | 0.697 | 13 | No |
| CD36 | IL-1a | cytokine | -0.275 | 0.363 | 0.697 | 13 | No |
| CD36 | IL-23 | cytokine | -0.269 | 0.373 | 0.697 | 13 | No |
| Ffar4 | Waist/length ratio | metabolic | 0.44 | 0.135 | 0.713 | 13 | No |
| Ffar4 | IL-17A | cytokine | 0.434 | 0.14 | 0.713 | 13 | No |
| Ffar4 | GM-CSF | cytokine | 0.418 | 0.157 | 0.713 | 13 | No |
| Ffar4 | Relative abdominal fat mass | metabolic | 0.401 | 0.176 | 0.713 | 13 | No |
| Ffar4 | IFN-g | cytokine | 0.341 | 0.255 | 0.713 | 13 | No |
| Ffar4 | Body Weight | metabolic | 0.319 | 0.289 | 0.713 | 13 | No |
| Ffar4 | IL-23 | cytokine | 0.319 | 0.289 | 0.713 | 13 | No |
| Ffar4 | IFN-b | cytokine | 0.319 | 0.289 | 0.713 | 13 | No |
| Ffar4 | IL-6R | receptor | 0.297 | 0.34 | 0.713 | 13 | No |
| Ffar4 | IL-10 | cytokine | 0.286 | 0.344 | 0.713 | 13 | No |
| Ffar4 | QUICKI | metabolic | -0.214 | 0.357 | 0.713 | 13 | No |
| Ffar4 | non-HDL | metabolic | 0.261 | 0.386 | 0.72 | 13 | No |
| Ffar4 | Leptin | metabolic | 0.231 | 0.448 | 0.737 | 13 | No |
| Ffar4 | MCP-1 | cytokine | 0.236 | 0.437 | 0.737 | 13 | No |
| FP number | TNF-R2 | receptor | 0.177 | 0.564 | 0.738 | 13 | No |
| FP area | TNF-R2 | receptor | 0.159 | 0.603 | 0.753 | 13 | No |
| Ffar4 | TNF-a | cytokine | 0.203 | 0.505 | 0.755 | 13 | No |
| Ffar4 | IL-1b | cytokine | 0.192 | 0.529 | 0.755 | 13 | No |
| Ffar4 | IL-1a | cytokine | 0.181 | 0.554 | 0.755 | 13 | No |
| Ffar4 | IL-6 | cytokine | 0.176 | 0.566 | 0.755 | 13 | No |
| Ffar4 | IL-27 | cytokine | 0.159 | 0.604 | 0.769 | 13 | No |
| GPR84 | IFN-g | cytokine | -0.429 | 0.171 | 0.784 | 13 | No |
| GPR84 | TNF-R2 | receptor | 0.385 | 0.212 | 0.784 | 13 | No |
| GPR84 | MCP-1 | cytokine | -0.302 | 0.34 | 0.784 | 13 | No |
| GPR84 | IL-1b | cytokine | -0.297 | 0.34 | 0.784 | 13 | No |
| GPR84 | Relative abdominal fat mass | metabolic | -0.291 | 0.34 | 0.784 | 13 | No |
| GPR84 | TNF-a | cytokine | -0.291 | 0.36 | 0.784 | 13 | No |
| GPR84 | Body Weight | metabolic | -0.286 | 0.317 | 0.784 | 13 | No |
| GPR84 | QUICKI | metabolic | 0.264 | 0.457 | 0.784 | 13 | No |
| GPR84 | IL-23 | cytokine | -0.231 | 0.49 | 0.784 | 13 | No |
| GPR84 | IL-1a | cytokine | -0.225 | 0.478 | 0.784 | 13 | No |
| GPR84 | GM-CSF | cytokine | -0.225 | 0.502 | 0.784 | 13 | No |
| GPR84 | IL-6 | cytokine | -0.203 | 0.513 | 0.784 | 13 | No |
| GPR84 | IL-17A | cytokine | -0.203 | 0.532 | 0.784 | 13 | No |
| GPR84 | non-HDL | metabolic | 0.201 | 0.5 | 0.784 | 13 | No |
| FP area | IFN-g | cytokine | -0.143 | 0.642 | 0.785 | 13 | No |
| FP number | IL-23 | cytokine | -0.143 | 0.64 | 0.785 | 13 | No |
| GPR84 | Leptin | metabolic | -0.132 | 0.659 | 0.8 | 13 | No |
| GPR84 | IFN-b | cytokine | 0.121 | 0.639 | 0.8 | 13 | No |
| GPR84 | TNF-R1 | receptor | -0.115 | 0.638 | 0.8 | 13 | No |
| GPR84 | Waist/length ratio | metabolic | -0.11 | 0.686 | 0.8 | 13 | No |
| CD36 | non-HDL | metabolic | -0.184 | 0.544 | 0.818 | 13 | No |
| CD36 | Waist/length ratio | metabolic | -0.17 | 0.579 | 0.818 | 13 | No |
| CD36 | IL-10 | cytokine | -0.17 | 0.579 | 0.818 | 13 | No |
| CD36 | GM-CSF | cytokine | -0.159 | 0.604 | 0.818 | 13 | No |
| FP area | GM-CSF | cytokine | -0.115 | 0.707 | 0.821 | 13 | No |
| Ffar4 | TNF-R1 | receptor | 0.093 | 0.747 | 0.836 | 13 | No |
| Tas1r2 | IL-6R | receptor | -0.049 | 0.845 | 0.845 | 13 | No |
| FP number | IL-27 | cytokine | -0.102 | 0.74 | 0.846 | 13 | No |
| FP number | GM-CSF | cytokine | 0.091 | 0.767 | 0.863 | 13 | No |
| FP number | TNF-R1 | receptor | 0.091 | 0.767 | 0.863 | 13 | No |
| GPR84 | IL-6R | receptor | 0.093 | 0.773 | 0.866 | 13 | No |
| FP area | TNF-R1 | receptor | 0.082 | 0.789 | 0.873 | 13 | No |
| Tas1r3 | IL-27 | cytokine | 0.055 | 0.863 | 0.878 | 13 | No |
| FP number | IFN-b | cytokine | -0.063 | 0.837 | 0.911 | 13 | No |
| Ffar1 | TNF-R1 | receptor | -0.473 | 0.109 | 0.92 | 13 | No |
| Ffar1 | Body Weight | metabolic | -0.341 | 0.255 | 0.92 | 13 | No |
| Ffar1 | IFN-g | cytokine | -0.324 | 0.28 | 0.92 | 13 | No |
| Ffar1 | QUICKI | metabolic | 0.308 | 0.347 | 0.92 | 13 | No |
| Ffar1 | Relative abdominal fat mass | metabolic | -0.302 | 0.315 | 0.92 | 13 | No |
| Ffar1 | Leptin | metabolic | -0.176 | 0.566 | 0.92 | 13 | No |
| Ffar1 | Waist/length ratio | metabolic | -0.181 | 0.554 | 0.92 | 13 | No |
| Ffar1 | GM-CSF | cytokine | -0.176 | 0.566 | 0.92 | 13 | No |
| Ffar1 | IL-17A | cytokine | -0.17 | 0.579 | 0.92 | 13 | No |
| Ffar1 | IL-1a | cytokine | -0.165 | 0.591 | 0.92 | 13 | No |
| Ffar1 | TNF-R2 | receptor | 0.165 | 0.606 | 0.92 | 13 | No |
| Ffar1 | MCP-1 | cytokine | -0.154 | 0.617 | 0.92 | 13 | No |
| Ffar1 | IL-1b | cytokine | -0.148 | 0.63 | 0.92 | 13 | No |
| Ffar1 | IFN-b | cytokine | 0.137 | 0.656 | 0.92 | 13 | No |
| Ffar1 | IL-23 | cytokine | -0.126 | 0.683 | 0.92 | 13 | No |
| Ffar1 | IL-6 | cytokine | -0.126 | 0.683 | 0.92 | 13 | No |
| Ffar1 | TNF-a | cytokine | -0.121 | 0.696 | 0.92 | 13 | No |
| Ffar1 | IL-27 | cytokine | 0.11 | 0.723 | 0.92 | 13 | No |
| Ffar4 | TNF-R2 | receptor | -0.055 | 0.874 | 0.925 | 13 | No |
| GPR84 | IL-10 | cytokine | 0.038 | 0.859 | 0.925 | 13 | No |
| Ffar1 | IL-6R | receptor | 0.044 | 0.845 | 0.938 | 13 | No |
| Ffar1 | IL-10 | cytokine | -0.033 | 0.92 | 0.938 | 13 | No |
| Ffar1 | non-HDL | metabolic | 0.03 | 0.924 | 0.938 | 13 | No |
| FP number | IL-6R | receptor | 0.041 | 0.893 | 0.94 | 13 | No |
| CD36 | IL-6R | receptor | 0.071 | 0.782 | 0.952 | 13 | No |
| GPR84 | IL-27 | cytokine | 0.005 | 0.939 | 0.974 | 13 | No |
| CD36 | IL-6 | cytokine | -0.038 | 0.906 | 1 | 13 | No |
| CD36 | IL-27 | cytokine | -0.022 | 0.949 | 1 | 13 | No |
| CD36 | IFN-b | cytokine | 0 | 1 | 1 | 13 | No |

*.*

***Table S6:*** *Edge Table Spleen.*

| **Anchor (taste/FP)** | **Partner** | **Partner Category** | **Spearman r** | **Raw p-value** | **FDR-adjusted p-value** | **n** | **In network (FDR<0.05, \|r\|>0.5)** |
| --- | --- | --- | --- | --- | --- | --- | --- |
| FP area | Waist/length ratio | metabolic | -0.786 | 0.00145 | 0.018 | 13 | Yes |
| FP number | QUICKI | metabolic | 0.761 | 0.0025 | 0.024 | 13 | Yes |
| Tas1r2 | Leptin | metabolic | -0.669 | 0.0125 | 0.024 | 13 | Yes |
| FP number | Waist/length ratio | metabolic | -0.737 | 0.00409 | 0.032 | 13 | Yes |
| FP area | Body Weight | metabolic | -0.725 | 0.00502 | 0.037 | 13 | Yes |
| FP number | Body Weight | metabolic | -0.612 | 0.0261 | 0.039 | 13 | Yes |
| Tas1r2 | Relative abdominal fat mass | metabolic | -0.619 | 0.0241 | 0.039 | 13 | Yes |
| FP number | Leptin | metabolic | -0.643 | 0.0178 | 0.045 | 13 | Yes |
| FP number | Relative abdominal fat mass | metabolic | -0.599 | 0.0307 | 0.048 | 13 | Yes |
| Tas1r2 | Waist/length ratio | metabolic | -0.641 | 0.0183 | 0.048 | 13 | Yes |
| Tas1r2 | Body Weight | metabolic | -0.702 | 0.00752 | 0.049 | 13 | Yes |
| Tas1r2 | IL-17A | cytokine | -0.68 | 0.014 | 0.084 | 13 | No |
| Tas1r2 | IL-1b | cytokine | -0.608 | 0.0276 | 0.112 | 13 | No |
| Tas1r2 | TNF-a | cytokine | -0.597 | 0.0313 | 0.122 | 13 | No |
| Tas1r2 | MCP-1 | cytokine | -0.591 | 0.0334 | 0.129 | 13 | No |
| FP number | IL-6 | cytokine | -0.563 | 0.0453 | 0.166 | 13 | No |
| Ffar1 | TNF-R2 | receptor | 0.555 | 0.049 | 0.176 | 13 | No |
| Tas1r2 | TNF-R1 | receptor | -0.541 | 0.056 | 0.193 | 13 | No |
| Tas1r3 | Leptin | metabolic | -0.538 | 0.0576 | 0.193 | 13 | No |
| Tas1r2 | IL-23 | cytokine | -0.53 | 0.0622 | 0.202 | 13 | No |
| FP area | Leptin | metabolic | -0.522 | 0.0673 | 0.212 | 13 | No |
| Tas1r2 | IL-1a | cytokine | -0.514 | 0.0725 | 0.225 | 13 | No |
| Ffar1 | Body Weight | metabolic | -0.505 | 0.078 | 0.233 | 13 | No |
| FP area | TNF-R1 | receptor | -0.5 | 0.0819 | 0.234 | 13 | No |
| FP number | non-HDL | metabolic | -0.504 | 0.079 | 0.234 | 13 | No |
| GPR84 | Waist/length ratio | metabolic | -0.489 | 0.0899 | 0.25 | 13 | No |
| GPR84 | TNF-R2 | receptor | 0.489 | 0.0899 | 0.25 | 13 | No |
| CD36 | TNF-R2 | receptor | 0.478 | 0.0985 | 0.267 | 13 | No |
| FP area | QUICKI | metabolic | 0.473 | 0.103 | 0.271 | 13 | No |
| Tas1r2 | QUICKI | metabolic | 0.475 | 0.101 | 0.271 | 13 | No |
| Tas1r2 | non-HDL | metabolic | -0.456 | 0.117 | 0.296 | 13 | No |
| Tas1r2 | IL-10 | cytokine | -0.459 | 0.115 | 0.296 | 13 | No |
| FP number | TNF-R1 | receptor | -0.452 | 0.121 | 0.299 | 13 | No |
| FP area | Relative abdominal fat mass | metabolic | -0.445 | 0.128 | 0.303 | 13 | No |
| Tas1r2 | IFN-g | cytokine | -0.436 | 0.136 | 0.314 | 13 | No |
| FP area | IL-17A | cytokine | -0.434 | 0.138 | 0.317 | 13 | No |
| Tas1r2 | TNF-R2 | receptor | 0.414 | 0.159 | 0.351 | 13 | No |
| Tas1r3 | IL-17A | cytokine | -0.412 | 0.162 | 0.351 | 13 | No |
| FP area | TNF-R2 | receptor | 0.407 | 0.168 | 0.359 | 13 | No |
| Tas1r3 | TNF-R2 | receptor | 0.401 | 0.174 | 0.365 | 13 | No |
| FP number | TNF-R2 | receptor | 0.4 | 0.176 | 0.366 | 13 | No |
| FP area | IL-1a | cytokine | -0.385 | 0.194 | 0.391 | 13 | No |
| Tas1r3 | IL-10 | cytokine | -0.379 | 0.201 | 0.397 | 13 | No |
| FP area | IL-6 | cytokine | -0.374 | 0.209 | 0.405 | 13 | No |
| Ffar1 | IFN-b | cytokine | 0.368 | 0.216 | 0.413 | 13 | No |
| FP number | IL-17A | cytokine | -0.359 | 0.229 | 0.434 | 13 | No |
| FP area | TNF-a | cytokine | -0.352 | 0.239 | 0.449 | 13 | No |
| Tas1r2 | IFN-b | cytokine | -0.343 | 0.252 | 0.471 | 13 | No |
| Ffar1 | GM-CSF | cytokine | 0.335 | 0.263 | 0.481 | 13 | No |
| FP area | MCP-1 | cytokine | -0.335 | 0.263 | 0.481 | 13 | No |
| GPR84 | Leptin | metabolic | -0.335 | 0.263 | 0.481 | 13 | No |
| GPR84 | Body Weight | metabolic | -0.335 | 0.263 | 0.481 | 13 | No |
| FP number | IL-1a | cytokine | -0.331 | 0.269 | 0.49 | 13 | No |
| GPR84 | GM-CSF | cytokine | 0.324 | 0.28 | 0.498 | 13 | No |
| GPR84 | TNF-R1 | receptor | -0.324 | 0.28 | 0.498 | 13 | No |
| Tas1r3 | IL-23 | cytokine | -0.324 | 0.28 | 0.498 | 13 | No |
| GPR84 | IFN-b | cytokine | 0.319 | 0.289 | 0.509 | 13 | No |
| Ffar1 | Waist/length ratio | metabolic | -0.308 | 0.306 | 0.529 | 13 | No |
| Ffar4 | IFN-b | cytokine | -0.308 | 0.306 | 0.529 | 13 | No |
| FP area | IL-1b | cytokine | -0.308 | 0.306 | 0.529 | 13 | No |
| Tas1r2 | IL-27 | cytokine | -0.309 | 0.304 | 0.529 | 13 | No |
| FP number | IL-10 | cytokine | -0.306 | 0.309 | 0.531 | 13 | No |
| CD36 | GM-CSF | cytokine | 0.302 | 0.316 | 0.532 | 13 | No |
| Ffar1 | IL-1b | cytokine | -0.302 | 0.316 | 0.532 | 13 | No |
| GPR84 | non-HDL | metabolic | -0.3 | 0.32 | 0.536 | 13 | No |
| Tas1r3 | Relative abdominal fat mass | metabolic | -0.297 | 0.325 | 0.536 | 13 | No |
| CD36 | non-HDL | metabolic | -0.283 | 0.348 | 0.57 | 13 | No |
| FP number | MCP-1 | cytokine | -0.281 | 0.352 | 0.572 | 13 | No |
| Tas1r3 | IL-1b | cytokine | -0.28 | 0.354 | 0.572 | 13 | No |
| FP number | TNF-a | cytokine | -0.279 | 0.357 | 0.575 | 13 | No |
| FP area | non-HDL | metabolic | -0.267 | 0.378 | 0.597 | 13 | No |
| FP area | IL-10 | cytokine | -0.264 | 0.384 | 0.597 | 13 | No |
| FP area | IFN-b | cytokine | -0.269 | 0.374 | 0.597 | 13 | No |
| GPR84 | Relative abdominal fat mass | metabolic | -0.264 | 0.384 | 0.597 | 13 | No |
| GPR84 | QUICKI | metabolic | 0.269 | 0.374 | 0.597 | 13 | No |
| CD36 | IL-1a | cytokine | -0.258 | 0.394 | 0.606 | 13 | No |
| Tas1r3 | MCP-1 | cytokine | -0.258 | 0.394 | 0.606 | 13 | No |
| CD36 | TNF-a | cytokine | -0.253 | 0.405 | 0.613 | 13 | No |
| CD36 | IFN-b | cytokine | 0.253 | 0.405 | 0.613 | 13 | No |
| Tas1r3 | IL-1a | cytokine | -0.253 | 0.405 | 0.613 | 13 | No |
| Ffar1 | TNF-a | cytokine | -0.247 | 0.415 | 0.622 | 13 | No |
| Ffar1 | IL-10 | cytokine | 0.247 | 0.415 | 0.622 | 13 | No |
| Ffar1 | TNF-R1 | receptor | -0.231 | 0.448 | 0.643 | 13 | No |
| FP area | IL-23 | cytokine | -0.231 | 0.448 | 0.643 | 13 | No |
| FP area | IL-27 | cytokine | -0.231 | 0.448 | 0.643 | 13 | No |
| FP number | IFN-g | cytokine | -0.234 | 0.441 | 0.643 | 13 | No |
| Tas1r3 | Body Weight | metabolic | -0.231 | 0.448 | 0.643 | 13 | No |
| Tas1r3 | TNF-a | cytokine | -0.231 | 0.448 | 0.643 | 13 | No |
| Tas1r3 | IL-27 | cytokine | -0.236 | 0.437 | 0.643 | 13 | No |
| Tas1r3 | TNF-R1 | receptor | 0.231 | 0.448 | 0.643 | 13 | No |
| Tas1r3 | IL-6 | cytokine | 0.225 | 0.459 | 0.652 | 13 | No |
| Ffar4 | Leptin | metabolic | -0.22 | 0.471 | 0.663 | 13 | No |
| Ffar4 | IL-27 | cytokine | -0.214 | 0.482 | 0.666 | 13 | No |
| GPR84 | IL-6 | cytokine | -0.214 | 0.482 | 0.666 | 13 | No |
| Tas1r2 | GM-CSF | cytokine | -0.215 | 0.48 | 0.666 | 13 | No |
| FP number | IL-1b | cytokine | -0.21 | 0.492 | 0.675 | 13 | No |
| FP area | IL-6R | receptor | -0.203 | 0.505 | 0.686 | 13 | No |
| Tas1r3 | Waist/length ratio | metabolic | -0.203 | 0.505 | 0.686 | 13 | No |
| CD36 | Leptin | metabolic | -0.198 | 0.517 | 0.691 | 13 | No |
| CD36 | Waist/length ratio | metabolic | -0.198 | 0.517 | 0.691 | 13 | No |
| CD36 | MCP-1 | cytokine | -0.198 | 0.517 | 0.691 | 13 | No |
| Ffar4 | IL-6 | cytokine | 0.198 | 0.517 | 0.691 | 13 | No |
| Ffar4 | TNF-R1 | receptor | 0.192 | 0.529 | 0.697 | 13 | No |
| Tas1r3 | IFN-b | cytokine | -0.192 | 0.529 | 0.697 | 13 | No |
| CD36 | IL-1b | cytokine | -0.187 | 0.541 | 0.709 | 13 | No |
| CD36 | IL-10 | cytokine | -0.181 | 0.553 | 0.715 | 13 | No |
| GPR84 | IL-17A | cytokine | -0.181 | 0.553 | 0.715 | 13 | No |
| CD36 | Body Weight | metabolic | -0.176 | 0.566 | 0.72 | 13 | No |
| CD36 | IL-17A | cytokine | -0.176 | 0.566 | 0.72 | 13 | No |
| CD36 | TNF-R1 | receptor | -0.176 | 0.566 | 0.72 | 13 | No |
| Ffar1 | non-HDL | metabolic | -0.176 | 0.565 | 0.72 | 13 | No |
| Ffar1 | MCP-1 | cytokine | -0.17 | 0.578 | 0.729 | 13 | No |
| Tas1r2 | IL-6 | cytokine | -0.171 | 0.576 | 0.729 | 13 | No |
| Ffar1 | IFN-g | cytokine | 0.165 | 0.59 | 0.738 | 13 | No |
| Ffar4 | IL-6R | receptor | -0.165 | 0.59 | 0.738 | 13 | No |
| CD36 | Relative abdominal fat mass | metabolic | -0.154 | 0.616 | 0.76 | 13 | No |
| Ffar1 | IL-1a | cytokine | -0.148 | 0.629 | 0.764 | 13 | No |
| Ffar4 | IL-10 | cytokine | -0.148 | 0.629 | 0.764 | 13 | No |
| GPR84 | IL-1a | cytokine | -0.148 | 0.629 | 0.764 | 13 | No |
| Ffar4 | TNF-R2 | receptor | -0.143 | 0.642 | 0.768 | 13 | No |
| FP area | IFN-g | cytokine | -0.143 | 0.642 | 0.768 | 13 | No |
| FP number | IL-23 | cytokine | -0.143 | 0.64 | 0.768 | 13 | No |
| GPR84 | TNF-a | cytokine | -0.143 | 0.642 | 0.768 | 13 | No |
| Tas1r3 | QUICKI | metabolic | 0.143 | 0.642 | 0.768 | 13 | No |
| CD36 | IL-6R | receptor | -0.137 | 0.655 | 0.772 | 13 | No |
| Tas1r3 | IL-6R | receptor | -0.137 | 0.655 | 0.772 | 13 | No |
| Ffar4 | IL-23 | cytokine | -0.132 | 0.668 | 0.779 | 13 | No |
| CD36 | IL-6 | cytokine | -0.126 | 0.681 | 0.785 | 13 | No |
| Ffar1 | IL-27 | cytokine | 0.126 | 0.681 | 0.785 | 13 | No |
| Tas1r3 | IFN-g | cytokine | -0.126 | 0.681 | 0.785 | 13 | No |
| CD36 | IL-27 | cytokine | -0.121 | 0.694 | 0.798 | 13 | No |
| Ffar1 | Leptin | metabolic | -0.115 | 0.707 | 0.807 | 13 | No |
| FP area | GM-CSF | cytokine | -0.115 | 0.707 | 0.807 | 13 | No |
| Tas1r2 | IL-6R | receptor | -0.105 | 0.733 | 0.83 | 13 | No |
| FP number | IL-27 | cytokine | -0.102 | 0.74 | 0.835 | 13 | No |
| CD36 | QUICKI | metabolic | 0.099 | 0.748 | 0.836 | 13 | No |
| CD36 | IL-23 | cytokine | -0.099 | 0.748 | 0.836 | 13 | No |
| GPR84 | MCP-1 | cytokine | -0.099 | 0.748 | 0.836 | 13 | No |
| Ffar4 | IFN-g | cytokine | 0.093 | 0.762 | 0.847 | 13 | No |
| FP number | GM-CSF | cytokine | 0.091 | 0.767 | 0.851 | 13 | No |
| Ffar1 | QUICKI | metabolic | 0.088 | 0.775 | 0.855 | 13 | No |
| Ffar4 | IL-17A | cytokine | -0.088 | 0.775 | 0.855 | 13 | No |
| Ffar4 | non-HDL | metabolic | -0.077 | 0.802 | 0.874 | 13 | No |
| Ffar4 | IL-1b | cytokine | 0.077 | 0.803 | 0.874 | 13 | No |
| Ffar4 | Body Weight | metabolic | 0.071 | 0.817 | 0.886 | 13 | No |
| FP number | IL-6R | receptor | -0.066 | 0.83 | 0.897 | 13 | No |
| FP number | IFN-b | cytokine | -0.063 | 0.837 | 0.901 | 13 | No |
| Ffar4 | Relative abdominal fat mass | metabolic | 0.06 | 0.845 | 0.902 | 13 | No |
| GPR84 | IL-27 | cytokine | -0.06 | 0.845 | 0.902 | 13 | No |
| Ffar1 | IL-23 | cytokine | 0.055 | 0.859 | 0.912 | 13 | No |
| Tas1r3 | non-HDL | metabolic | -0.055 | 0.858 | 0.912 | 13 | No |
| Ffar4 | TNF-a | cytokine | 0.049 | 0.873 | 0.923 | 13 | No |
| GPR84 | IL-10 | cytokine | -0.049 | 0.873 | 0.923 | 13 | No |
| GPR84 | IL-23 | cytokine | -0.044 | 0.887 | 0.93 | 13 | No |
| GPR84 | IFN-g | cytokine | 0.044 | 0.887 | 0.93 | 13 | No |
| GPR84 | IL-1b | cytokine | -0.044 | 0.887 | 0.93 | 13 | No |
| Ffar1 | IL-6R | receptor | 0.027 | 0.929 | 0.952 | 13 | No |
| Ffar4 | Waist/length ratio | metabolic | 0.027 | 0.929 | 0.952 | 13 | No |
| Ffar4 | MCP-1 | cytokine | 0.027 | 0.929 | 0.952 | 13 | No |
| GPR84 | IL-6R | receptor | 0.027 | 0.929 | 0.952 | 13 | No |
| Tas1r3 | GM-CSF | cytokine | 0.027 | 0.929 | 0.952 | 13 | No |
| Ffar4 | IL-1a | cytokine | 0.022 | 0.943 | 0.96 | 13 | No |
| CD36 | IFN-g | cytokine | -0.016 | 0.957 | 0.972 | 13 | No |
| Ffar1 | Relative abdominal fat mass | metabolic | -0.011 | 0.972 | 0.981 | 13 | No |
| Ffar4 | QUICKI | metabolic | -0.011 | 0.972 | 0.981 | 13 | No |
| Ffar1 | IL-6 | cytokine | -0.005 | 0.986 | 0.988 | 13 | No |
| Ffar1 | IL-17A | cytokine | -0.005 | 0.986 | 0.988 | 13 | No |
| Ffar4 | GM-CSF | cytokine | 0.005 | 0.986 | 0.988 | 13 | No |

***Table S7.*** Housekeeping gene-normalized gene-expression values for all measured receptors across the tested tissues. Values are depicted as mean (min-max).

|  | CV | | Jejunum | |
| --- | --- | --- | --- | --- |
|  | CTRL | CAF | CTRL | CAF |
| IL-6R | 0,141  (0,000-0,2490) | 0,255  (0,2040-0,3235) | 0,0002407  (0-0,0006181) | 0,0002807  (0-0,0006988) |
| TNF-R1 | 0,5780  (0,1358-1,171) | 0,4433  (0,4014-0,4798) | 0,01431  (0,006863-0,02266) | 0,02008  (0,01535-0,02669) |
| TNF-R2 | 0,7431  (0,09328-2,015) | 0,05603  (0,03373-0,09115) | 0,002667  (0,0004899-0,004580) | 0,001086  (0,0003670-0,002087) |
| Tas1r2 | 0,2307  (0,06929-0,7279) | 0,04909  (0,002151-0,08292) | n.d. | n.d. |
| Tas1r3 | 0,04591  (0-0,08307) | 0,07777  (0,01611-0,1194) | 0,0001235  (2,648e-005-0,0004361) | 8,185e-005  (0,000-0,0003010) |
| CD36 | 1,273  (0,1323-3,234) | 0,03919  (0,01402-0,08739) | 0,005743  (0,001497-0,01022) | 0,001261  (0,0001765-0,003674) |
| Ffar1 | 0,3480  (0,04486-0,8096) | 0,01227  (0,003319-0,03439) | 0,002913  (0,0004609-0,004661) | 0,0009845  (0,0001966-0,002320) |
| GPR84 | 0,5955  (0,04920-1,697) | 0,01170  (0,001983-0,03861) | 0,002104  (0,0004256-0,004026) | 0,0004800  (0,0001653-0,0009725) |
| Ffar4 | 0,1886  (0,02027-0,6588) | 0,01848  (0,01288-0,03394) | 0,002239  (0,0008180-0,003502) | 0,001987  (0,001070-0,003405) |
|  |  | | | |
|  | Stomach | | Spleen | |
|  | CTRL | CAF | CTRL | CAF |
| IL-6R | 0,002  (0,0007089-0,004045) | 0,003031  (0,0007364-0,008662) | 0,01070  (0,003132-0,01530) | 0,01107  (0,006932-0,01390) |
| TNF-R1 | 0,01028  (0,007957-0,01273) | 0,009636  (0,006287-0,01118) | 0,009263  (0,004086-0,01588) | 0,01217  (0,01062-0,01514) |
| TNF-R2 | 0,003441  (0,002623-0,005974) | 0,002630  (0,001887-0,003444) | 0,04106  (0,02396-0,07905) | 0,02300  (0,01931-0,02639) |
| Tas1r2 | 0,0001433  (3,770e-006-0,0005194) | 0,0002394  (0,0001371-0,0004503) | 0,0004722  (3,586e-006-0,001757) | 2,465e-005  (0,000-0,0001176) |
| Tas1r3 | 7,982e-005  (0-0,0002550) | 0,0001253  (4,509e-005-0,0002603) | 0,0004774  (5,182e-005-0,001206) | 0,0003412  (1,342e-005-0,0006794) |
| CD36 | 0,002265  (0,0003869-0,006631) | 0,001013  (0,0005325-0,001410) | 0,02385  (0,001292-0,09865) | 0,002289  (0,0002962-0,004239) |
| Ffar1 | 0,001022  (0,0001117-0,003238) | 0,0005155  (0,0004009-0,0007215) | 0,01898  (0,003467-0,06600) | 0,005523  (0,003226-0,009900) |
| GPR84 | 0,0005559  (0,0001539-0,001407) | 0,0002924  (0,0001785-0,0004094) | 0,00851  (0,0002888- 0,03709) | 0,0009608  (0,0001292-0,002520) |
| Ffar4 | 0,0002669  (9,472e-005-0,0005291) | 0,0005170  (0,0001829-0,0007999) | 0,001026  (0,0004300-0,002032) | 0,0008896  (0,0006308-0,001407) |

***S8. Network Analysis Stomach***


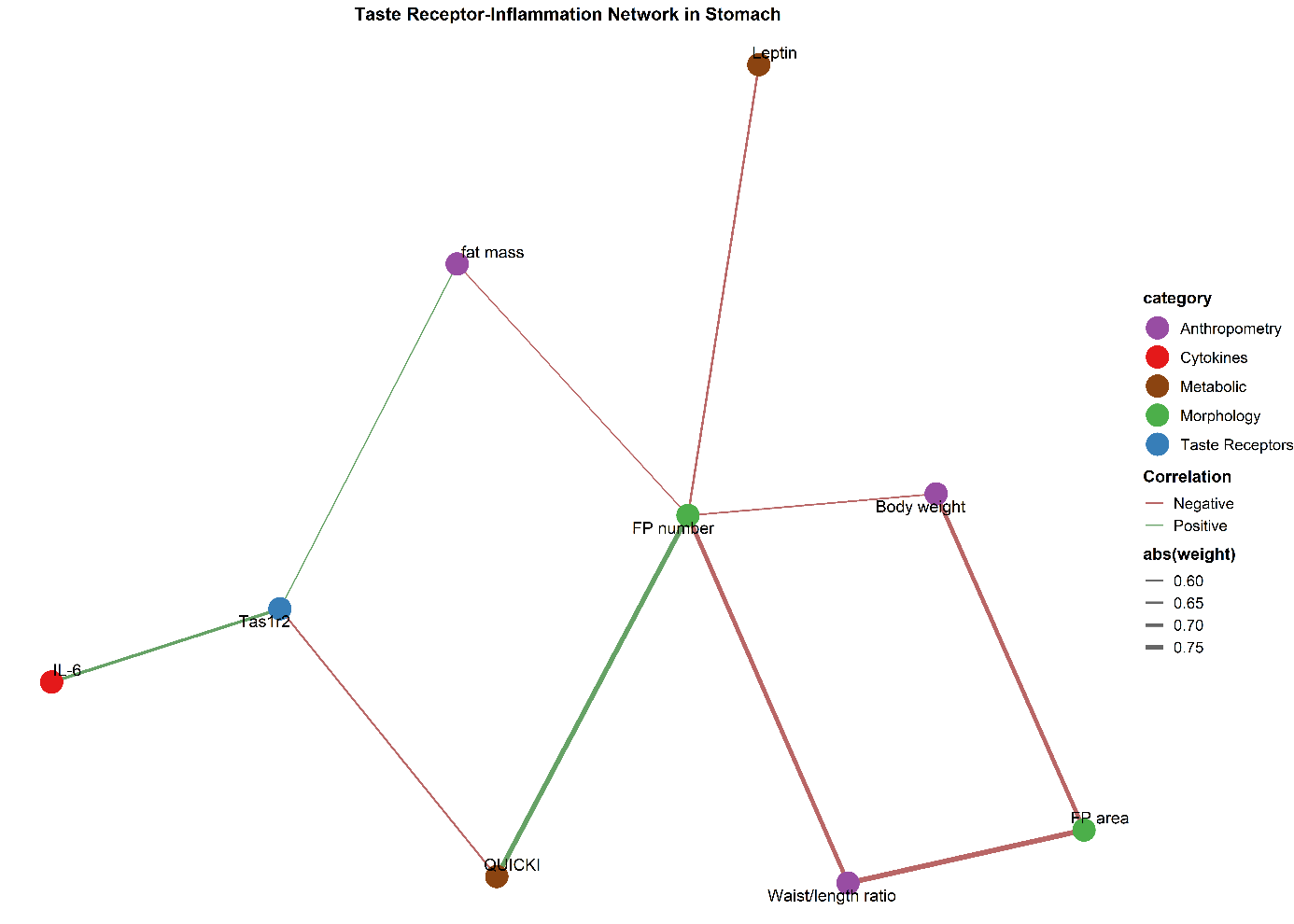


***S3.*** *Network Analysis of taste marker (taste receptors in stomach and fungiform papillae). Depicted taste (Tas1r2, Tas1r3, CD36, Ffar1, GPR84, Ffar4) and inflammatory receptors (IL-6R, TNF-R1, TNF-R2) were analyzed in stomach. The network shows correlations with │r│ > 0.5 and FDR-adjusted p < 0.05, positive correlations are displayed in green, negative correlations in red, line thickness indicates correlation strength. Pairwise correlations between all variables were calculated using Spearman's rank correlation coefficient and p-values were corrected using false discovery rate.*

***S9. Network Analysis Spleen***


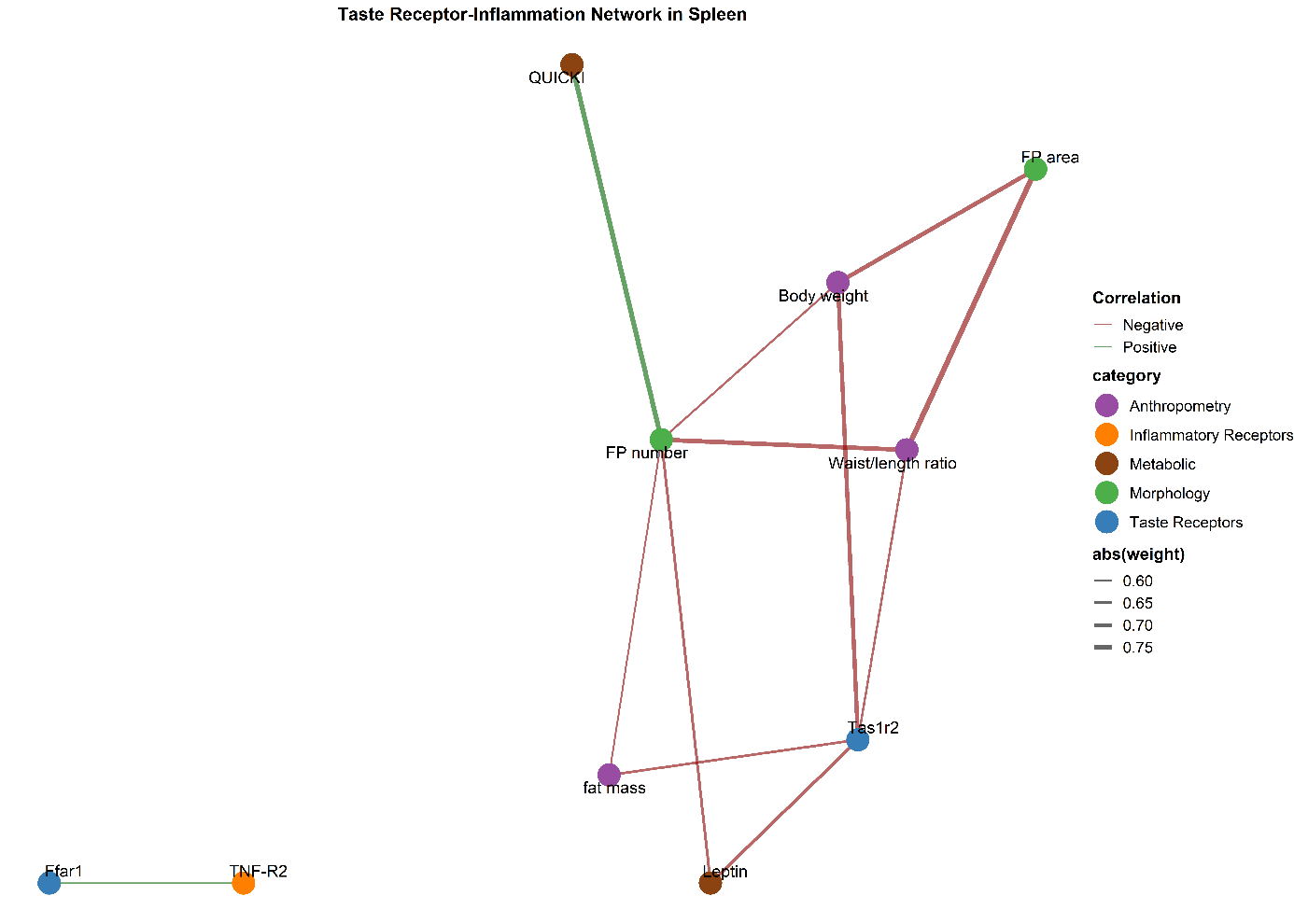


***S4.*** *Network Analysis of taste marker (taste receptors in spleen and fungiform papillae). Depicted taste (Tas1r2, Tas1r3, CD36, Ffar1, GPR84, Ffar4) and inflammatory receptors (IL-6R, TNF-R1, TNF-R2) were analyzed in spleen. The network shows correlations with│ r│ > 0.5 and FDR-adjusted p < 0.05, positive correlations are displayed in green, negative correlations in red, line thickness indicates correlation strength. Pairwise correlations between all variables were calculated using Spearman's rank correlation coefficient and p-values were corrected using false discovery rate.*
